# HIF-1α is a key regulator in potentiating suppressor activity and limiting the microbicidal capacity of MDSC-like cells during visceral leishmaniasis

**DOI:** 10.1371/journal.ppat.1006616

**Published:** 2017-09-11

**Authors:** Akil Hammami, Belma Melda Abidin, Tania Charpentier, Aymeric Fabié, Annie-Pier Duguay, Krista M. Heinonen, Simona Stäger

**Affiliations:** INRS-Institut Armand-Frappier and Center for Host-Parasite interactions, 531 Boulevard des Prairies, Laval (QC), Canada; National Institute of Health, UNITED STATES

## Abstract

*Leishmania donovani* is known to induce myelopoiesis and to dramatically increase extramedullary myelopoiesis. This results in splenomegaly, which is then accompanied by disruption of the splenic microarchitecture, a chronic inflammatory environment, and immunosuppression. Chronically inflamed tissues are typically hypoxic. The role of hypoxia on myeloid cell functions during visceral leishmaniasis has not yet been studied. Here we show that *L*. *donovani* promotes the output from the bone marrow of monocytes with a regulatory phenotype that function as safe targets for the parasite. We also demonstrate that splenic myeloid cells acquire MDSC-like function in a HIF-1α-dependent manner. HIF-1α is also involved in driving the polarization towards M2-like macrophages and rendering intermediate stage monocytes more susceptible to *L*. *donovani* infection. Our results suggest that HIF-1α is a major player in the establishment of chronic *Leishmania* infection and is crucial for enhancing immunosuppressive functions and lowering leishmanicidal capacity of myeloid cells.

## Author summary

The protozoan parasite *Leishmania donovani* causes chronic infection in the spleen, which is accompanied by a chronic inflammatory environment, an enlargement of the organ, and immunosuppression. The environment of chronically inflamed tissues is characterized by low oxygen levels and tissue disruption, which induce the expression of the transcription factor HIF-1α in all cells. The kinetics of monocyte production and differentiation in the bone marrow and the spleen, and the role of hypoxia in myeloid cell functions during visceral leishmaniasis have not yet been studied. Here we show that *L*. *donovani* promotes the output from the bone marrow of monocytes with a regulatory phenotype that function as safe targets for the parasite. We also demonstrate that HIF-1α potentiates inhibitory functions of myeloid cells and is involved in driving the polarization towards M2-like macrophages and rendering them more susceptible to *L*. *donovani* infection. Our results suggest that HIF-1α is a major player in the establishment of chronic *Leishmania* infection and is crucial for enhancing immunosuppressive functions and lowering leishmanicidal capacity of myeloid cells.

## Introduction

Elimination of intracellular pathogens requires the induction of pro-inflammatory cytokines and cytotoxic molecules secretion. Unfortunately, this process also leads to local tissue disruption and inflammation. Inflamed tissues represent a challenging microenvironment, characterized by hypoxia, acidosis and hypoglycemia. This microenvironment typically causes the stabilization of the transcription factor HIF-1α, the master regulator of the response to hypoxia [1, 2]. HIF-1α has pleiotropic functions aimed at protecting tissues from injury and helping cells to adapt to a difficult microenvironment. However, stabilization of HIF-1α in some cells of the immune system, such as myeloid cells, may also have unwanted consequences. For instance, HIF-1α is responsible for the polarization towards the M2-like phenotype of tumor-associated macrophages (TAM) [3], promoting therefore tumor growth. HIF-1α was also shown to enhance function and differentiation of myeloid derived suppressor cells (MDSC) in the tumor microenvironment [4]. Moreover, we have reported that HIF-1α stabilization in dendritic cells inhibited their function and consequently limited the expansion of protective CD8 T cell responses during experimental visceral leishmaniasis (VL) [5].

The HIF-pathway is also exploited by some pathogens for their replication and/or survival inside the host’s cell [6–9]. One example of such a pathogen is *Leishmania*. The protozoan parasite *Leishmania* is the causative agent of leishmaniasis, a disease with multiple clinical manifestations ranging from self-healing cutaneous and mucocutaneous lesions to potentially lethal visceral infections. The promastigote form of the parasite is transmitted to the host by a sandfly vector. Once inside the host, promastigotes transform into amastigotes. Macrophages are the main target cells of the parasite. However, to survive inside macrophages, *Leishmania* needs to attenuate their microbicidal potential [10]. One of the many strategies is the stabilization of HIF-1α [11], which appears to be essential for the survival of the promastigote form inside the cell [6, 11]. HIF-1α stabilization can occur following massive infiltration by pro-inflammatory cells in the tissue and/or as a consequence of pathogen invasion. These two phenomena are associated with increased oxygen consumption, which causes a local hypoxic environment [12]. During visceral leishmaniasis, HIF-1α stabilization is also induced in uninfected cells by the inflammatory environment and appears to hamper DC functions [5]. To date, the role of HIF-1α in other myeloid cells during in vivo *Leishmania* infections has not yet been explored.

Dendritic cells and neutrophils have been extensively studied in various models of leishmaniasis; however, the contribution of monocytes to susceptibility and/or resistance to infection is still unclear. The early literature proposes a possible role of “undifferentiated macrophage-granulocytes” as safe targets for *Leishmania*, contributing therefore to disease susceptibility [13]. Passos et al. [14] demonstrate that intermediate monocytes are involved in mediating immunopathology in patients infected with *L*. *braziliensis*. Another study reports the upregulation of A_2B_ adenosine receptors on human monocytes and the association of this upregulation with pathogenicity in patients exposed to *L*. *donovani* [15]. In contrast, monocyte-derived DC appear to be essential for priming protective Th1 responses in *L*. *major* infected mice [16] and classical monocytes are thought to be able to kill *L*. *major* [17] and *L*. *braziliensis* via reactive oxygen species [18].

In this study, we wanted to investigate the role of HIF-1α stabilization in myeloid cells, particularly monocytes, during experimental chronic VL. We found that myeloid cells are increasingly recruited to the spleen during chronic infection. Splenic myeloid cells upregulate HIF-1α and display HIF-1α-dependent inhibitory function on protective Th1 responses. Moreover, HIF-1α limits their leishmanicidal functions and regulates the differentiation and output of inflammatory monocytes from the bone marrow.

## Results

### Myeloid cells, particularly Ly6C^hi^ and Ly6C^lo/int^ monocytes, accumulate in the spleen of *L*. *donovani* infected mice over the course of infection

**Fig 1:**
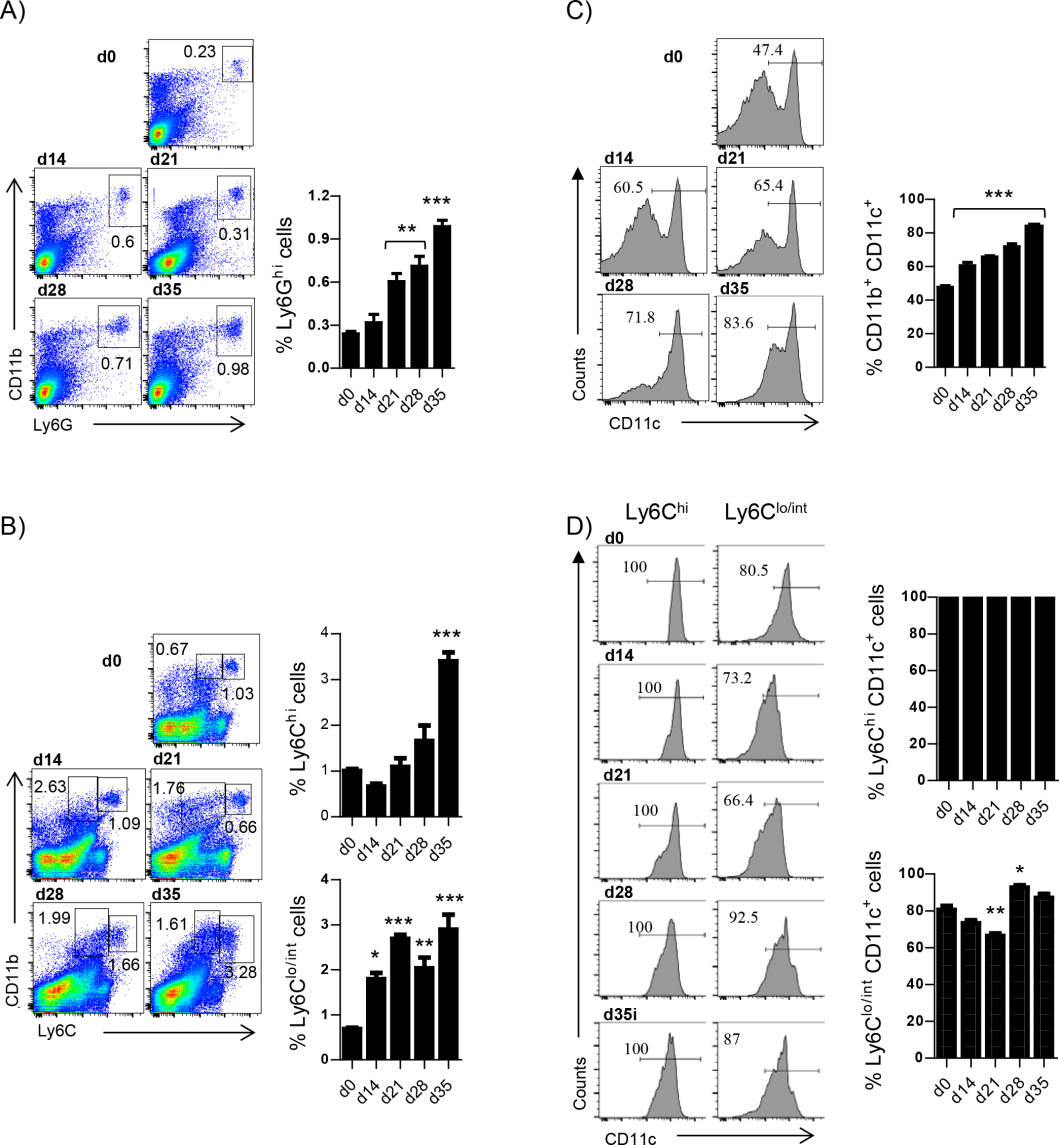
Myeloid cells, particularly Ly6C^hi^ and Ly6C^lo/int^ monocytes, accumulate in the spleen of *L*. *donovani* infected mice over the course of infection. Mice were infected with *L*. *donovani* and sacrificed at various time points after infection. Neutrophils were excluded from all analysis involving monocytes. **(A)** Representative FACS plots depicting the gating strategy used to identify neutrophils (left) and percentage of neutrophils in the spleen of infected mice (right).**(B)** Gating strategy used to identify Ly6C^+^ monocytes (left) and percentage of splenic Ly6C^hi^ (upper graph) and Ly6C^lo/int^ (lower graph) monocytes. **(C)** Percentage of CD11c^+^ myeloid cells. **(D)** Percentage of CD11c^+^ Ly6C^+^ (left histogram raw and upper graph) and Ly6C^lo/int^ (right histogram raw and lower graph) monocytes. All data represent mean ± SEM of one of 4 independent experiments, n = 4.

The literature about the role of monocytes during experimental visceral leishmaniasis is scarce. Hence, we wanted to have a full picture of the monocytes and neutrophils recruitment kinetics to the spleen over the course of experimental *L*. *donovani* infection, before assessing the role of HIF-1α in splenic myeloid cells. We first monitored the frequency of CD11b^hi^ Ly6G^hi^ neutrophils. As shown in Fig 1A, the percentage of neutrophils present in the spleen gradually increased during the first 4 weeks of infection. Similarly to neutrophils, Ly6C^hi^ monocytes were increasingly recruited to the spleen over the course of infection (Fig 1B). In contrast, the frequency of Ly6C^lo/int^ monocytes did not vary substantially as disease progressed (Fig 1B). Interestingly, the two monocyte populations were less easily distinguishable during the chronic phase of infection.

We next examined whether splenic myeloid cells expressed CD11c at various time points of infection. At d14 p.i. about 55% of all CD11b^+^ cells in the spleen expressed CD11c; the percentage of CD11c^+^ cells increased over the course of infection and at d35p.i. 80% of the splenic CD11b^+^ cells were also CD11c^+^ (Fig 1C). As expected, all Ly6C^hi^ monocytes were CD11c^+^ and about 85% of the Ly6C^lo/int^ monocytes expressed CD11c (Fig 1D).

Because LysM-specific HIF-1α-deficient mice are not a good model to study the role of HIF-1α in monocytes/macrophages in the spleen [19] and the vast majority of splenic CD11b^+^ cells during VL were CD11c^+^, we decided to use CD11c-specific HIF-1α deficient mice [5] to investigate the role of HIF-1α in myeloid cells, particularly monocytes, during chronic VL. To note, neutrophils did not express CD11c, hence they are HIF-sufficient in both groups of mice.

### HIF-1α- deficient mice in CD11c^+^ cells show increased frequency and numbers of inflammatory monocytes in the spleen

**Fig 2:**
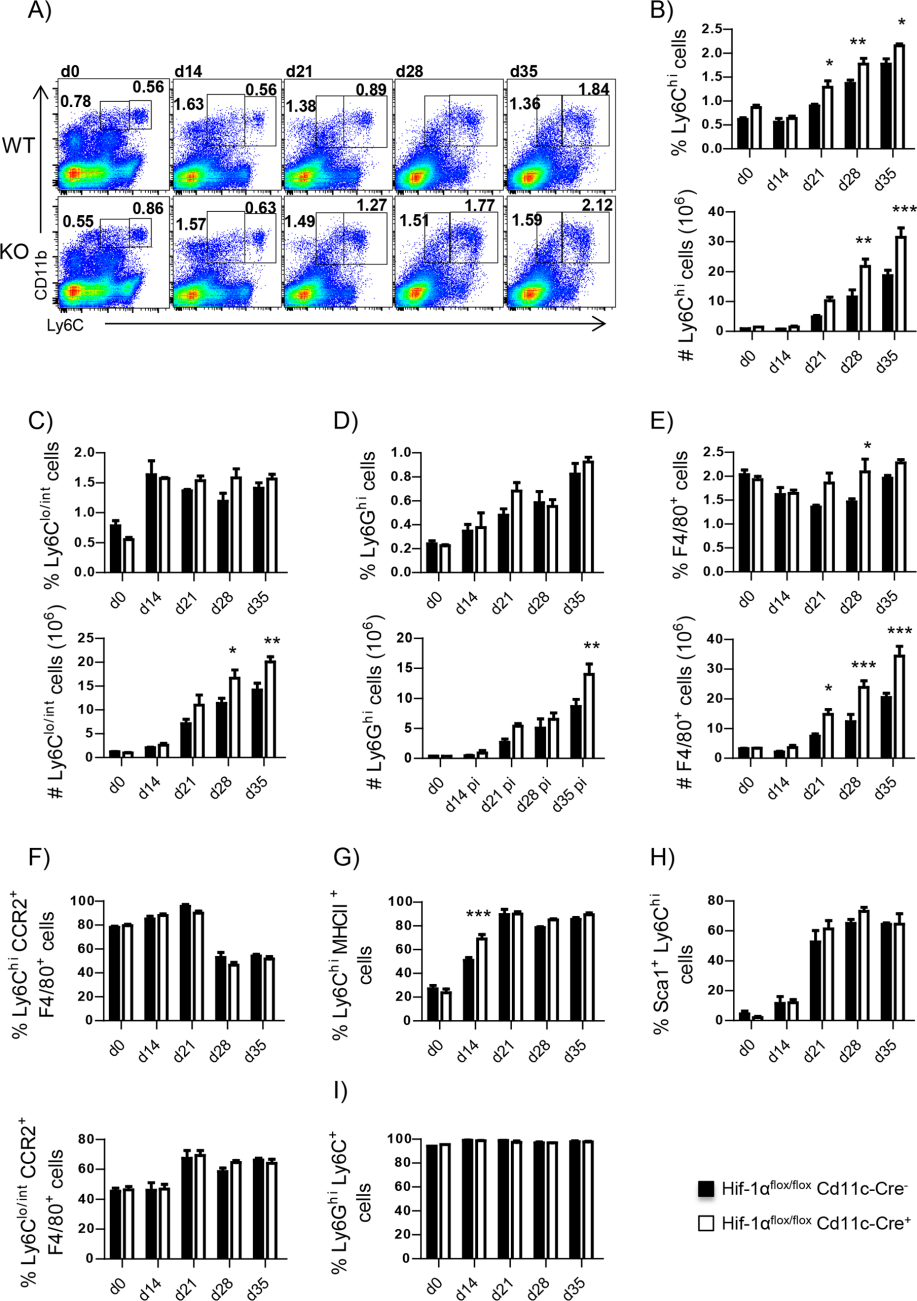
HIF-1α- deficient mice in CD11c^+^ cells show increased frequency and numbers of inflammatory monocytes in the spleen. *Hif*^*flox/flox*^-*Cd11c-Cre*^*+*^ and *Cre*^*-*^ mice were infected with *L*. *donovani* and sacrificed at various time point of infection. Neutrophils were excluded from all analysis involving monocytes. **(A)** Representative FACS plots depicting L6C^+^ monocytes in *Cre*^*-*^ (upper graphs) and *Cre*^*+*^ (lower graphs) mice over the course of infection. **(B-F)** Percentage (upper graph) and absolute numbers (lower graph) of splenic Ly6C^hi^ monocytes **(B)**, Ly6C^lo/int^ monocytes **(C)**, Ly6G^+^neutrophils **(D)**, F4/80^+^cells **(E)**, CCR2^+^F4/80^+^ Ly6C^hi^ monocytes **(F)**. **(G)** Percentage of splenic MHCII^+^ Ly6C^hi^ monocytes. **(H)** Percentage of splenic Sca-1^+^ Ly6C^hi^ monocytes. **(I)** Percentage of splenic Ly6C^+^ Ly6G^+^ neutrophils. All data represent mean ± SEM of one of 4 independent experiments, n = 4. * denotes *p*<0.05.

We have previously reported that *Hif*^*flox/flox*^–*Cd11c-Cre*^*+*^ mice are highly resistant to *L*. *donovani* infection ([5] and S1A Fig). During the acute phase of infection, HIF-1α impairs dendritic cell functions and limits CD8 T cell expansion [5]. At this stage of disease, parasite clearance in these mice is mainly CD8 T cell-dependent [5]; however, it is still unclear how these mice control *L*. *donovani* growth during chronic VL, when CD8 T cells are exhausted [20]. CD8^+^ dendritic cells are thought to be responsible for CD8 T cell cross-priming [21]. These DC subpopulation, unlike CD4^+^ DCs, mainly expresses DNGR1 (S1B Fig) and thus directly descends from DC precursors rather than being monocyte-derived [22]. Hence, we decided to extend our investigation on the role of HIF-1α to other myeloid cells, particularly monocytes and monocytes-derived cells. Because monocytes contribute to parasite clearance in other models of leishmaniasis [16–18], we first compared the recruitment of monocytes to the spleen in *Hif*^*flox/flox*^–*Cd11c-Cre*^*+*^ mice (HIF-1α-deficient) and their *Cre*^*-*^ littermates (HIF-1α-sufficient) at various time points of infection. Before, though, we monitored HIF-1α expression in purified CD11b^+^ cells from both mouse groups to confirm that HIF-1α was indeed deleted in *Hif*^*flox/flox*^–*Cd11c-Cre*^*+*^ myeloid cells (S2A and S2B Fig). As observed in C57BL/6 mice, the frequency and the number of Ly6C^hi^ monocytes increased over the course of infection in the *Cre*^*-*^and *Cre*^*+*^ group (Fig 2A and 2B). However, a significantly higher number of inflammatory monocytes was present in the spleen of *Cre*^*+*^ mice. Non-classical Ly6C^lo/int^ monocytes (Fig 2A and 2C) and neutrophils (Fig 2D and S3A Fig) displayed similar frequencies in both mouse groups, but cell numbers were higher in *Cre*^*+*^ mice, reflecting a slightly more pronounced splenomegaly in HIF-1α conditional knockouts. Similar results were obtained when we examined F4/80 expression in myeloid cells (Fig 2E and S3B Fig).

Next, we further characterized splenic monocytes by monitoring the expression of CCR2 and F4/80, and MHCII on Ly6C^lo/int^ and Ly6C^hi^ cells. 85% of Ly6C^hi^ monocytes co-expressed CCR2 and F4/80 at d14 and 21p.i.; the frequency then decreased to 50% at later time points of infection (Fig 2F and S4A Fig). No differences were observed between HIF-1α-sufficient and deficient monocytes. The frequency of CCR2^+^F4/80^+^ Ly6C^lo/int^ monocytes steadily increased over the course of infection to reach a plateau of about 70% at d21p.i. (Fig 2F and S4B Fig) in both groups of mice. These monocytes possibly represent an intermediate stage in the differentiation process towards macrophages.

Surprisingly, 50% of *Cre*^*-*^ Ly6C^hi^ monocytes were positive for MHCII; by d21p.i., the frequency of MHCII^+^ inflammatory monocytes increased to 80–90% and was maintained at this level during chronic infection (Fig 2G and S4C Fig). The percentage of MHCII^+^ Ly6C^hi^ monocytes was slightly higher in HIF-1α-deficient mice at d14, d28, and d35p.i. Recently, Ly6C^hi^ monocytes with a regulatory phenotype have been described [23]. These monocytes are induced by IFNγ in the bone marrow and express MHCII and Sca-1. Hence, we assessed Sca-1 expression on monocytes. From d21 p.i. on, the majority of the Ly6C^hi^ monocytes expressed Sca-1, suggesting that inflammatory monocytes may also display a regulatory phenotype during chronic VL (Fig 2H).

Based on our surface marker analysis, splenic monocytes resembled monocytic myeloid-derived suppressor cells (M-MDSC) [24] and/or monocyte with a regulatory phenotype [23]. The other known subset of MDSC originates from polymorphonucleated cells (PMN-MDSC) and is characterized by the co-expression of Ly6G and Ly6C (Ly6G^+^Ly6C^lo^) [24].To determine whether PMN-MDSC were also present in the spleen of *L*. *donovani* infected mice, we monitored the surface expression of Ly6C on CD11b^hi^Ly6G^hi^ neutrophils. 100% of the neutrophils were Ly6C^+^ already at d14p.i. (Fig 2I and S4D Fig); Ly6C expression was maintained during the chronic phase. This suggests that neutrophils express similar markers to PMN-MDSC and could potentially exhibit immune suppressive properties.

### HIF-1α induces an M2-like phenotype and limits leishmanicidal capacity in myeloid cells

**Fig 3:**
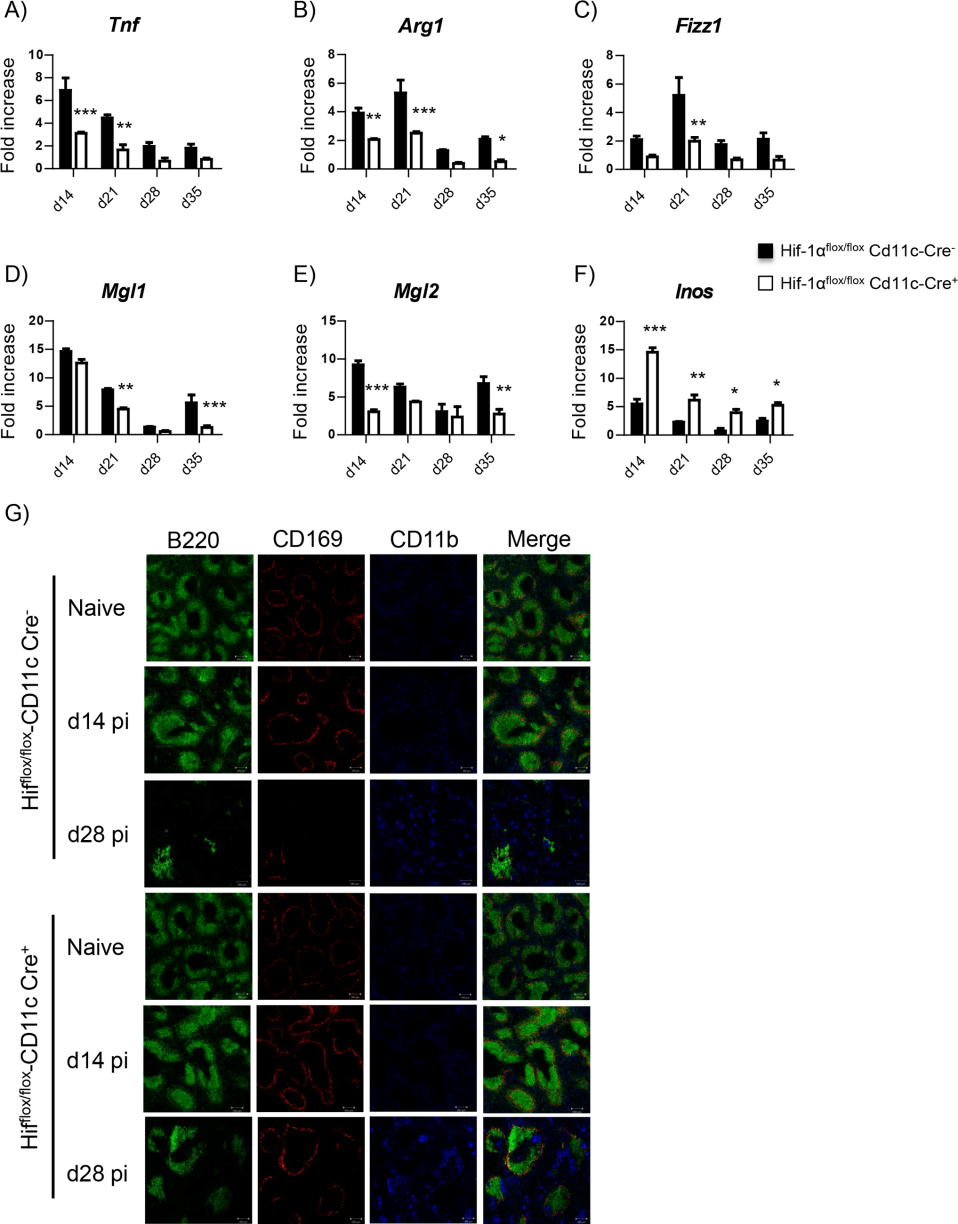
HIF-1α induces an M2-like phenotype and limits leishmanicidal capacity in myeloid cells. *Hif*^*flox/flox*^-*Cd11c-Cre*^*+*^ and *Cre*^*-*^ mice were infected with *L*. *donovani* and sacrificed at various time point of infection. **(A—F)** Real-time PCR analysis of mRNA expression levels in splenic CD11b^+^ cells purified from infected mice at various time points after infection for **(A)** *Tnf*, **(B)** *Arg1*, **(C)** *Fizz1*, **(D)** *Mgl1*, **(E)** *Mgl2*, and **(F)** *iNOS*. (**G**) Immonohistochemical analysis of splenic sections from naïve and infected mice at d14 and 28 p.i.; CD169 (red), B220 (green), CD11b (blue); magnification: 10x.

In the following, we sought to characterize myeloid cell function. To this end, CD11b^+^ cells were purified from the spleen of infected *Cre*^*-*^ and *Cre*^*+*^ mice at various time points of infection; the expression of several genes was assessed by qPCR. Interestingly, CD11b^+^ cells from *Hif*^*flox/flox*^–*Cd11c-Cre*^*+*^ mice showed a lower expression of TNF (Fig 3A), arginase (Fig 3B), Fizz1 (Fig 3C), Mgl1, and Mgl2 (Fig 3D and 3E); in contrast, they expressed higher iNOS mRNA levels (Fig 3F). Hence, HIF-1α seems to sustain the differentiation towards the M2-like macrophage subtype.

Between d14 and 21 p.i., splenic stromal cells are killed by excessive TNF production [25]; consequently, the splenic microarchitecture is altered [26]. Disruption of the microarchitecture is typically accompanied by the progressive loss of B cell Germinal Centers [27]. Interestingly, the splenic microarchitecture in infected *Cre*^*+*^ mice appeared to be more intact than in the *Cre-* controls at d28 p.i. (Fig 3G). This may be a consequence of the lower TNF production by myeloid cells (Fig 3A). Notably, myeloid cells (Fig 3G, blue) were increasingly present in the splenic red pulp of infected mice after d14 p.i.

**Fig 4:**
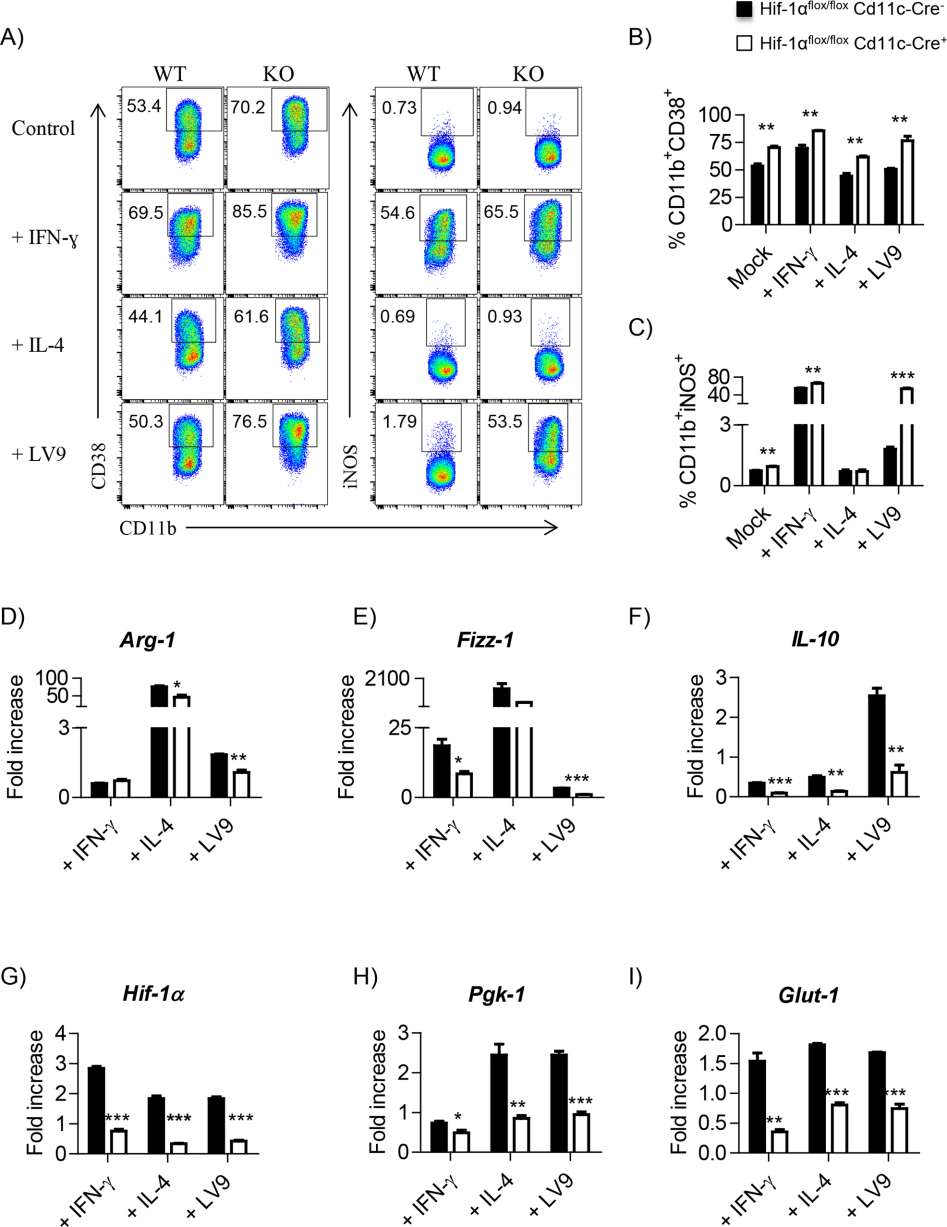
*L*. *donovani* amastigotes strongly induce iNOS production in HIF-1α-deficient BMM. Macrophages were derived for six days from the bone marrow of naïve *Hif*^*flox/flox*^-*Cd11c-Cre*^*+*^ and *Cre*^*-*^ mice. Cells were then activated with IFNγ or IL-4, or infected *L*. *donovani*. Polarization and infection were monitored for 24h. **(A)** Representative FACS plot for macrophages expressing CD38^+^ (left panels) and *iNOS*^*+*^ *(*right panels) in *Hif-1α*^*flox/flox*^*Cd11c-cre*^*-*^ (WT) and *Cre*^*+*^ (KO) mice. Frequency of **(B)** CD38^+^ and **(C)** iNOS^+^ in different polarization conditions and following infection. **(D—I)** Real-time PCR analysis of mRNA expression levels in in vitro polarized and infected BMM. **(D)** *Arg1*, **(E)** *Fizz1*, **(F)** *Il10*, **(G)** *Hif1α*, **(H)** *Pgk1* and **(I)** *Glut1*. All data represent mean ± SEM, n = 3. * denotes *p*<0.05, ** denotes *p*<0.01, and *** denotes p<0.001.

HIF-1α has been reported to promote iNOS expression [28–30]. Hence, we were surprised to observe an increase in iNOS mRNA levels in myeloid cells from infected *Cre*^*+*^ mice (Fig 3F). To verify our in vivo observation, we infected HIF-1α-sufficient and deficient bone marrow-derived macrophages (BMM) with *L*. *donovani* amastigotes and analyzed iNOS production by flow cytometry. CD38 was used as an M1 marker. As expected, stimulation of BMM with IFNγ increased the percentage of CD38^+^ cells (Fig 4A and 4B) and the production of iNOS (Fig 4A and 4C), which was slightly higher in HIF-1α-deficient cells. In contrast, treatment with IL-4 failed to promote iNOS (Fig 4C) and reduced the frequency of CD38^+^ cells (Fig 4B), independently from the presence or absence of HIF-1α. However, when we infected BMM with *L*. *donovani* amastigotes, a dramatic increase in iNOS production was observed in HIF-1α deficient BMM but not in HIF-1α sufficient cells (Fig 4A and 4C), confirming our in vivo observation (Fig 3F). We also analyzed the expression of M2 markers Arg-1 (Fig 4D), Fizz-1 (Fig 4E), and IL-10 (Fig 4F). A slight decrease in the levels of Arg-1 and Fizz-1 mRNA was detected in *Cre*^*+*^ compared to *Cre*^*-*^ cells; moreover, IL-10 mRNA was not upregulated in HIF-1α deficient BMM following infection with *L*. *donovani*. To be sure that HIF-1α was indeed deleted in BMM from conditional knockouts, we assessed the expression of HIF-1α and two HIF-1α downstream targets, Pgk-1 and Glut-1 in cytokine-treated and infected BMM. HIF-1α (Fig 4G), Pgk-1 (Fig 4H) and Glut-1 (Fig 4I) were not induced in HIF-1α deficient BMM following *L*. *donovani* infection or cytokine treatment, suggesting that recombination occurred in BMM from *Cre*^*+*^ mice.

**Fig 5:**
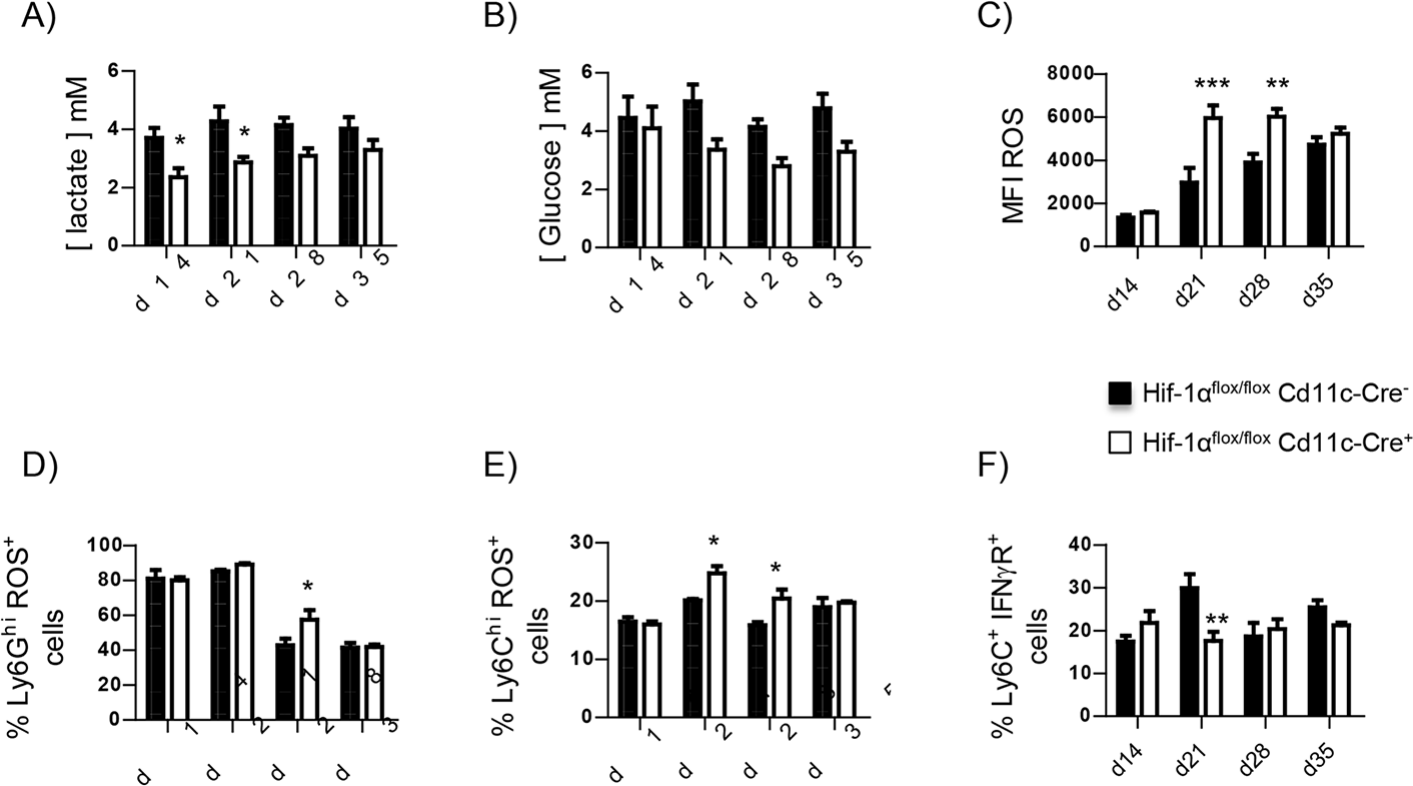
HIF-1α governs glucose metabolism in *L*. *donovani* infected splenocytes. *Hif*^*flox/flox*^-*Cd11c-Cre*^*+*^ and *Cre*^*-*^ mice were infected with *L*. *donovani* and sacrificed at various time point of infection. **(A)** Intracellular lactate concentration and **(B)** intracellular glucose concentration in splenocytes from infected mice at various time points of infection. **(C)** Mean fluorescence intensity of ROS expression in splenocytes from infected mice over the course of infection. **(D)** Percentage of ROS^+^ neutrophils. **(E)** Percentage of ROS^+^ Ly6C^hi^ monocytes. (**F**) Frequency of CD11b^hi^Ly6C^+^ splenocytes expressing IFNγR. All data represent mean ± SEM of one of 4 independent experiments, n = 4. * denotes *p*<0.05, ** denotes *p*<0.01, and *** denotes p<0.001

Because HIF-1α is known to regulate cell metabolism, we next measured intracellular lactate (Fig 5A) and glucose levels (Fig 5B). Interestingly, HIF-1α-sufficient myeloid cells had a higher intracellular lactate concentration compared to HIF-1α-deficient cells (Fig 5A), reflecting the metabolic switch towards anaerobic glycolysis [31, 32]. *Cre*^*-*^ cells also displayed a slightly higher intracellular glucose concentration (Fig 5B). We also assessed the production of reactive oxygen species (ROS), which are typically not generated by M2 macrophages [32]. HIF-1α–deficient splenocytes expressed higher levels of ROS (Fig 5C and S5A Fig). Neutrophils (Fig 5D and S5B Fig) and inflammatory monocytes (Fig 5E and S5C Fig) lacking HIF-1α contributed to this difference.

To rule out the possibility that myeloid cells acquired an M2-like phenotype because of higher levels of IFNγ present in the environment, we assessed the expression of the INFγ receptor by FACS. As shown in Fig 5F, the frequency of CD11b^hi^Ly6C^+^ cells expressing IFNγR was similar in both groups of mice, with exception of d21 p.i., when the expression was lower in *Cre*^*+*^ mice.

Taken together, these results suggest that HIF-1α may be involved in the differentiation towards macrophages with an M2-like phenotype, which is unable to kill *Leishmania* [31, 33].

### HIF-1α enhances the inhibitory functions of myeloid cells during chronic VL

**Fig 6:**
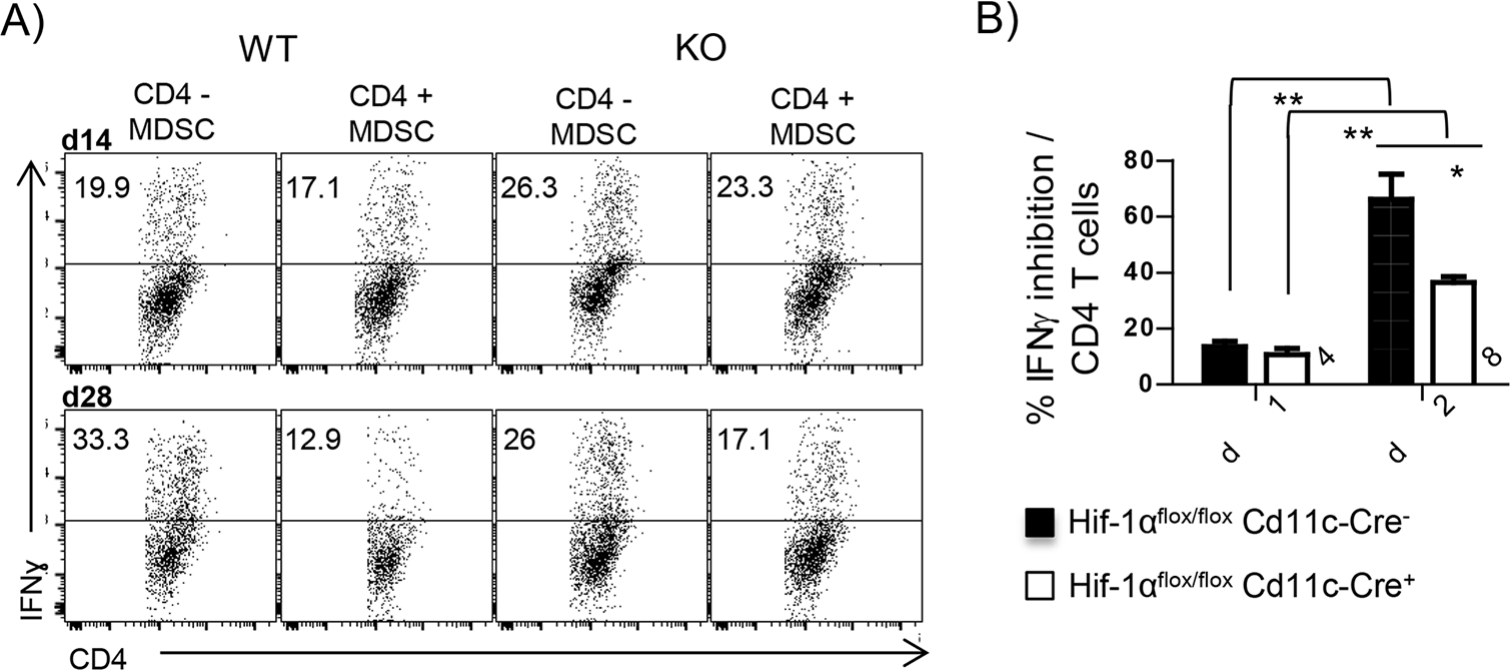
HIF-1α enhances the inhibitory functions of myeloid cells during chronic VL. Naïve CD4 T cells were stimulated with plate bound αCD3 and αCD28 in the presence of rIL-12. **(A)** Representative FACS plots showing IFNγ production by CD4 T cells co-incubated or not with CD11b^+^ cells purified from the spleen of *L*. *donovani* infected mice. **(B)** Percentage of inhibition of IFNγ production calculated as described in the material and methods section. All data represent mean ± SEM of one of 3 independent experiments, n = 3. * denotes *p*<0.05 and ** denotes *p*<0.01.

We next investigated the inhibitory potential of splenic myeloid cells. CD11b^+^ cells were purified from the spleen of *L*. *donovani* infected mice at d14 and 28 p.i. and co-cultured at a 1:1 ratio with naïve CD4 T cells stimulated with plate-bound anti-CD3 and with anti-CD28 and rIL-12. Myeloid cells purified from infected *Cre*^*-*^ mice at d14 p.i. only slightly inhibited the differentiation towards IFNγ-producing CD4 T cells (Fig 6A and 6B); a similar result was obtained with HIF-1α-deficient myeloid cells purified at the same time. Remarkably, d28 p.i. CD11b^+^ cells from infected HIF-1α sufficient mice strongly inhibited Th1 differentiation (Fig 6A and 6B); a significantly lower degree of inhibition was observed in samples containing d28 p.i. HIF-1α-deficient myeloid cells.

Taken together, our results suggest that myeloid cells purified during chronic infection inhibit T cell responses, implying that these cells are phenotypically and functionally similar to MDSC. This inhibitory function requires HIF-1α. Thus, this transcription factor is not only involved in attenuating the leishmanicidal capacity of myeloid cells, but also in enhancing their inhibitory function.

### HIF-1α deficient intermediate stage monocytes are more resistant to *L*. *donovani* infection under hypoxic conditions

**Fig 7:**
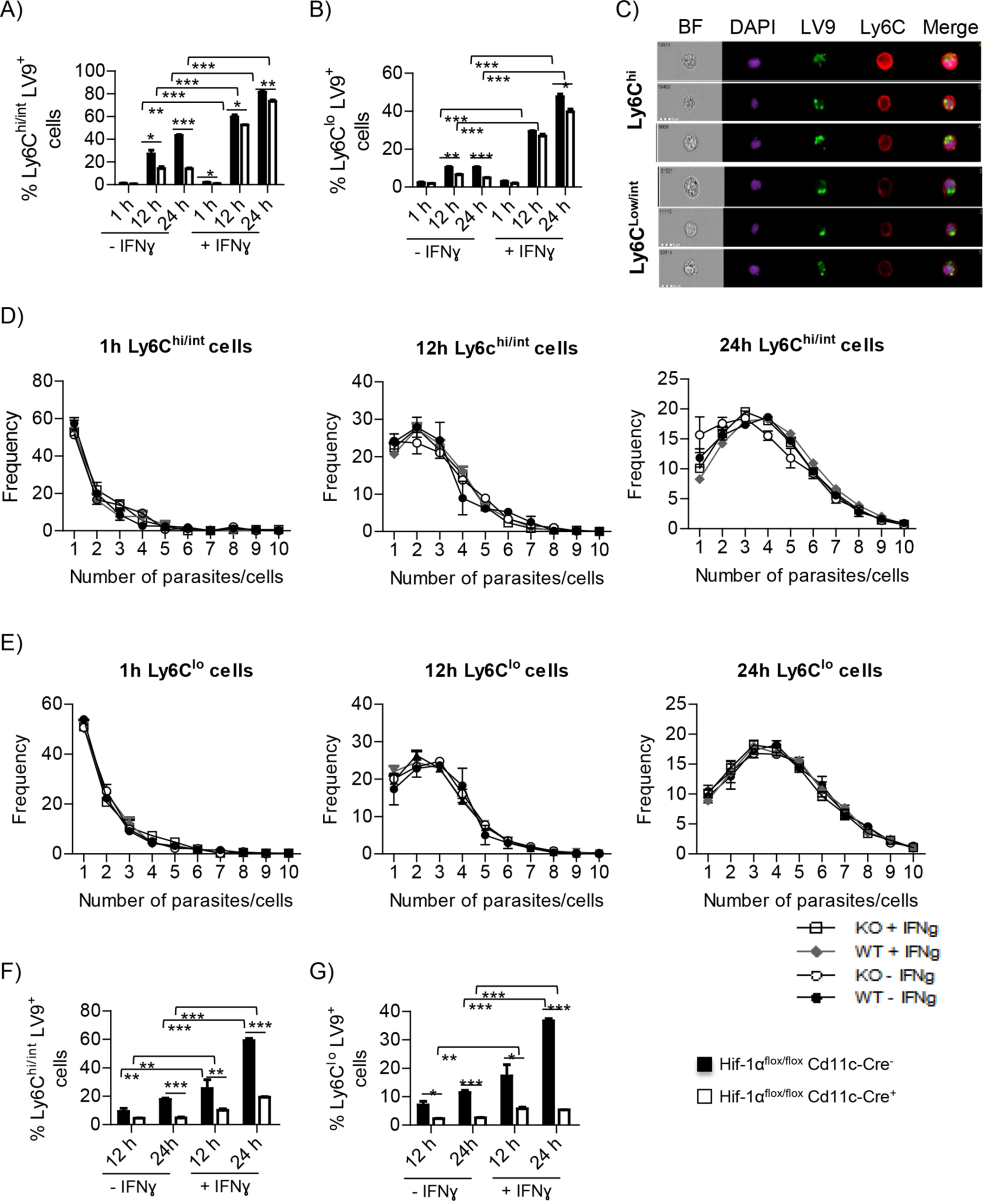
HIF-1α deficient intermediate stage monocytes are more resistant to *L*. *donovani* infection under hypoxic conditions. Monocytes were derived for three days from the bone marrow of naïve *Hif*^*flox/flox*^-*Cd11c-Cre*^*+*^ and *Cre*^*-*^ as described in the material and method section. M-CSF was then removed from the medium and cells were then infected with fluorescently-labelled *L*. *donovani* amastigotes prior to activation or not with IFNγ. The infection was monitored for 24h. **(A-B)** Percentage of infected Ly6C^hi^ **(A)** and Ly6C^lo/int^ **(B)** monocytes. **(C)** Examples of ImageStream analysis; pictures illustrate: nucleus (purple), parasites (green), Ly6C (red). **(D-E)** ImageStream analysis of numbers of parasites per cells in Ly6C^hi^ **(D)** and Ly6C^lo/int^ **(E)** monocytes at 1h (left graph), 12h (middle graph) and 24h (right graph). **(F-G)** Monocytes were treated as described above, but M-CSF was left in the medium. **(F)** Percentage of Ly6C^hi^ and **(G)** Ly6C^lo/int^ monocytes infected with *L*. *donovani*. All data represent mean ± SEM of one of 2 independent experiments, n = 3. * denotes *p*<0.05, ** denotes *p*<0.01, and *** denotes p<0.001

To determine whether HIF-1α-deficient monocytes were more resistant to infection by *L*. *donovani*, we infected bone marrow-derived monocytes in vitro with fluorescently labelled amastigotes and monitored the infection for 24h by FACS and Image Stream. Monocytes were either activated or not with IFNγ 2h prior to infection; cells were kept under hypoxic conditions at all time to mimic the bone marrow [34] and the splenic environment (S6A Fig). We first confirmed that *Cre*^*+*^ cells had a reduced HIF-1α expression (S6B Fig). About 25–30% of HIF-1α–sufficient Ly6C^hi/int^ monocytes contained parasites after 12h of infection; at 24h, 40–45% of the cells harbored parasites (Fig 7A). Interestingly, when monocytes were exposed to IFNγ prior to infection, the percentage of parasitized cells dramatically increased to 60% at 12h and 80% at 24h of infection (Fig 7A and S6C Fig). This is probably due to the fact that IFNγ induces regulatory Ly6C^hi/int^ monocytes [23] and that these may be more permissive for *L*. *donovani* amastigotes. In contrast, HIF-1α-deficient inflammatory monocytes were significantly less parasitized at 12 and 24h in the absence of IFNγ (Fig 7A and S6C Fig); as for their HIF-1α-sufficient counterparts, the addition of IFNγ dramatically increased the rate of infection (Fig 6A), suggesting that HIF-1α is not involved in inducing regulatory monocytes. Nevertheless, IFNγ-pulsed HIF-1α-deficient Ly6C^hi/int^ monocytes were slightly more resistant to infection than wild type inflammatory monocytes. Similar results were obtained when we analyzed the degree of infection of Ly6C^lo^ monocytes (Fig 7B and S6D Fig). We next determined whether the number of parasites per cell was equal in both groups of mice using the ImageStream technology (examples of analysis are depicted on Fig 7C). As shown in Fig 7D and 7E, no major differences were observed in the percentage of cells harboring various numbers of parasites between HIF-1α-deficient and HIF-1α-sufficient monocytes.

Because HIF-1α is upregulated during the differentiation of monocytes to macrophages [35], we were intrigued to know whether HIF-1α would play a role in resistance/susceptibility to infection if we kept M-CSF in the medium during infection with *L*. *donovani* to allow differentiation into macrophages. Surprisingly, we observed a highly significant reduction in the percentage of Ly6C^hi^ cells harboring parasites in HIF-1α-deficient monocytes (Fig 7F and S7A Fig), independently whether they were pulsed or not with IFNγ. Similar results were obtained when we analyzed the rate of infection in Ly6C^lo/int^ monocytes (Fig 7G and S7B Fig). Because *Leishmania* amastigotes survival in macrophages is not impaired in the absence of HIF-1α [5, 36], this suggests that HIF-1α mainly increases the susceptibility to infection of transitional forms of monocytes/macrophages.

### CD11c-specific HIF-1α-knockout mice produce more monocyte’s progenitors and display enhanced output of inflammatory monocytes in the bone marrow

**Fig 8:**
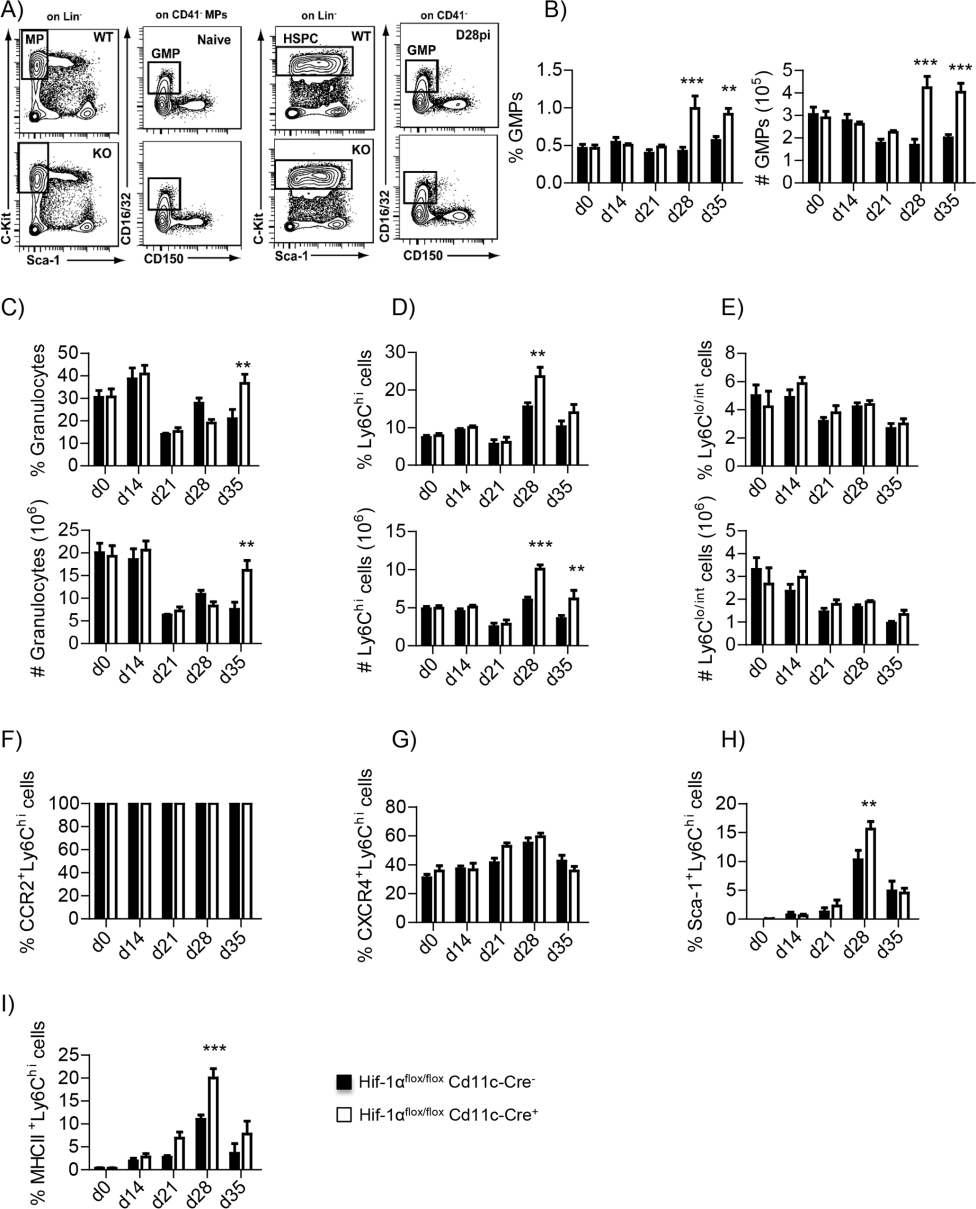
CD11c-specific HIF-1α-knockout mice produce more monocyte’s progenitors and display enhanced output of inflammatory monocytes in the bone marrow. *Hif*^*flox/flox*^-*Cd11c*-*Cre*^*+*^ and *Cre*^*-*^ mice were infected with *L*. *donovani* and sacrificed at various time point of infection. **(A)** Gating strategy to identify GMPs in the bone marrow of *L*.*donovani* infected mice. **(B)** Frequency (left graph) and absolute numbers (right graph) of GMPs in the bone marrow of mice over the course of infection. **(C-E)** Frequency (upper graph) and absolute numbers (lower graph) of granulocytes **(C)**, Ly6C^hi^ monocytes**(D),** Ly6C^lo/int^ monocytes **(E)** in the bone marrow of infected mice. **(F-G)** Percentage of CCR2^+^ **(F)** and CXCR4^+^ **(G)** Ly6C^hi^ monocytes in the bone marrow. **(H)** Percentage of Sca-1^+^ Ly6C^hi^ monocytes in the bone marrow. **(I)** Percentage of MHCII^+^ Ly6C^hi^ monocytes in the bone marrow. All data represent mean ± SEM of one of 4 independent experiments, n = 4. * denotes *p*<0.05.

We previously showed that *L*. *donovani* induces the proliferation of myeloid-biased hematopoietic stem progenitor cell (HSPCs) in the bone marrow, and that this infection-induced change in hematopoiesis could promote parasite expansion [37]. Furthermore, our myeloid cells resembled MDSC and MDSC are derived from the myeloid lineage and are a result of altered hematopoiesis during cancer or chronic infections. We therefore investigated the state of myeloid progenitors in the bone marrow of *L*. *donovani Cre*^*+*^ and *Cre*^*-*^ mice. Steady state bone marrow myeloid progenitors were gated as negative for all lineage markers, positive for c-kit and negative for Sca-1; they were then subdivided according to the expression of CD41, CD150, CD16/32 to define granulocyte-monocyte progenitors (GMP) as CD16/32^hi^ CD150^-^ cells (Fig 8A, left panels). Interestingly, along with the increase in HSPCs expansion, we detected a shift in Sca-1 expression on HSPCs (Fig 8A, right panels). In order to include emergency GMPs, known as Sca-1^+^ GMPs [38], GMPs were identified as Sca-1^+/-^Lin-c-Kit^+^CD16/32^hi^CD150^-^ CD41^-^ (Fig 8A, right panels). In agreement with our observations in the spleen, HIF-1α conditional knockout mice had a higher frequency and numbers of GMPs (Fig 8B) at day 28 and 35 p.i. This was paralleled by a significantly enhanced output of granulocytes at d35 p.i. (Fig 8C) and Ly6C^hi^ monocytes (Fig 8D) at day 28 and 35 p.i. No differences were observed for the Ly6C^lo/-^ monocytes (Fig 8E). It is important to note that the granulocyte and monocyte output does not increase during the first 3 weeks of *L*. *donovani* infection, suggesting that the bone marrow, like the spleen, does not majorly react to the infection during the acute phase. Furthermore, the absence of difference in the bone marrow was expected as the progenitor cells were HIF-1α sufficient (no CD11c expression).

To assess possible differences in the capacity to exit the bone marrow between monocytes from *Hif* ^*flox/flox*^-*Cd11c-Cre*^*+*^ and *Cre*^*-*^ mice, we monitored CCR2 and CXCR4 expression. No differences were found between both groups of mice (Fig 8F and 8G). When we monitored the surface expression of Sca-1 and MHCII, we could find a significant difference in terms of percentage of Sca-1 expression at d28 p.i. in infected *Cre*^*+*^ compared to *Cre*^*-*^ mice (Fig 8H). A similar result was obtained when we assessed MHCII expression (Fig 8I), suggesting that the inflammatory stimuli received by Ly6C^hi^ monocytes were similar in both groups of mice. To note, Sca-1 expression increased at d21 p.i. to peak at d28p.i. and gradually decreased thereafter (Fig 8H), showing a different expression kinetics than on splenic Ly6C^hi^ monocytes (Fig 2H).

### HIF-1α expression in CD11c^+^ cells exacerbates infection in the bone marrow

**Fig 9:**
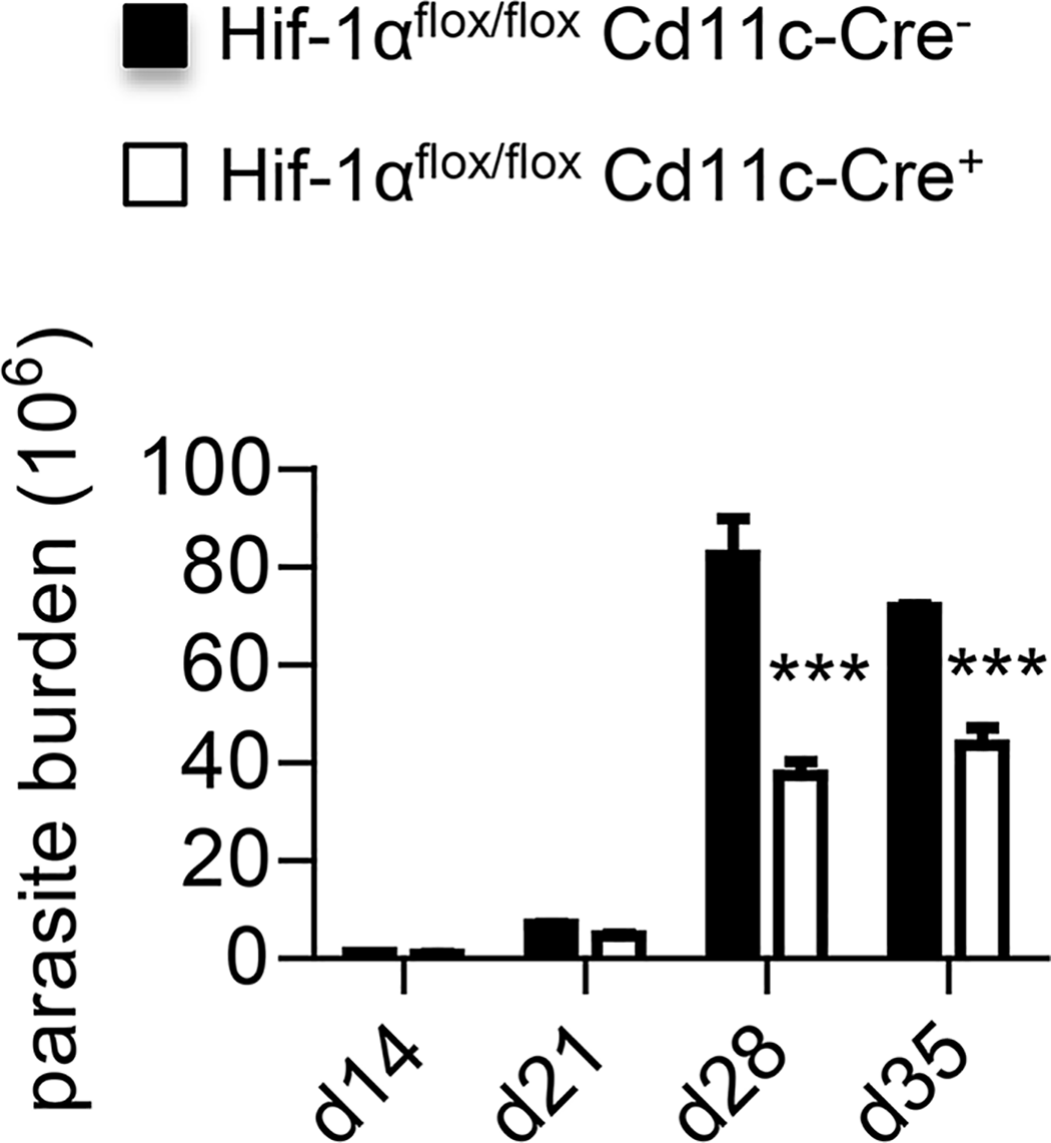
HIF-1α expression in CD11c^+^ cells exacerbates infection in the bone marrow. *Hif*^*flox/flox*^-*Cd11c-Cre*^*+*^ and *Cre*^*-*^ mice were infected with *L*. *donovani* and sacrificed at various time point of infection. Graph shows the number of parasites present in one tibia and one femur of each infected mouse over the course of infection. All data represent mean ± SEM of one of 4 independent experiments, n = 4. * denotes *p*<0.05 and ** denotes *p*<0.01.

HIF-1α CD11c-conditional knockouts are highly resistant to *L*. *donovani* infection in the spleen ([5] and S1 Fig), but the hepatic parasites number does not significantly differ from that of the control mice [5]. Hence, we were curious to assess the parasite burden in the bone marrow of our HIF-1α conditional knockouts. In agreement with the literature [39], we observed a dramatic increase in the number of parasite after 3 weeks of infection in the *cre*^*-*^ control group (Fig 9). The parasite burden peaked at d28 p.i. in *Cre*^*+*^ mice as well, but to a lesser extent. Indeed, we observed more than 50% reduction in conditional knockout mice, suggesting that HIF-1α expression in CD11c^+^ cells is detrimental to the outcome of *L*. *donovani* infection in the bone marrow as well.

## Discussion

The hematopoietic system rapidly increases myeloid cell output to fight pathogens. Emergency myelopoiesis also occurs during visceral leishmaniasis [37]. Our study shows that emergency myelopiesis in the context of VL results in the generation of regulatory monocytes that are more permissive to *Leishmania* parasites. Moreover, myeloid cells acquire an MDSC-like phenotype in the spleen and display HIF-1α-dependent T cell inhibitory functions. HIF-1α also drove the polarization towards M2-like macrophages and rendered intermediate stage monocytes more susceptible to *L*. *donovani* infection. Our results suggest that HIF-1α largely contributes to the establishment of chronic *Leishmania* infection by enhancing immunosuppressive functions and lowering leishmanicidal capacity of myeloid cells.

During experimental VL, myeloid cell generation is enhanced in the bone marrow by a parasite- induced boost in GM-CSF production [40]; extramedullary myelopoiesis in the spleen is also dramatically increased [39]. We observed a significant increase in GMPs in the bone marrow, which mainly resulted in a selective enhanced output of Ly6C^hi^ monocytes (see also [37]). Interestingly, inflammatory monocytes started upregulating Sca-1 and MHCII, two markers associated with regulatory monocytes [23], at day 21 after infection. In *L*. *donovani* infected mice, CD4^+^ Th1 responses typically peak between day 21 and 28 of infection. Hence, it is possible that Th1 cells prime Ly6C^hi^ monocytes for regulatory functions in the bone marrow. It is not surprising, thus, that IFNγ-primed monocytes are more permissive to in vitro *L*. *donovani* infection. Acquisition of regulatory functions was not dependent on HIF-1α, since *Hif*^*flox/flox*^–*CD11c-Cre*^-^ monocytes showed a similar infection rate to *Cre*^*+*^ cells. HIF-dependent inhibitory functions were first acquired during differentiation into macrophages. Adaptation to hypoxia in human monocytes was shown to be governed by NFƙB1 and not HIF-1α [41]. However, during the differentiation towards macrophages, HIF-1α translocates from the cytosol to the nucleus, changing therefore the adaptation mechanism to hypoxic conditions [41]. This may also apply to IFNγ-primed mouse monocytes. Hence, *Leishmania* induces an increased output of inflammatory monocytes in the bone marrow that acquire HIF-1α-independent regulatory functions prior to egress and represent therefore “safe targets” for the parasite, as postulated by an early study by Mirkovich et al. [13].

Increased rate of medullary or extramedullary myelopoiesis often leads to the induction of MDSC. MDSC are a heterogeneous population of various intermediate stages of myeloid cell differentiation that are best defined by their characteristic T cell-inhibitory activity. Based on the current classification [24], the majority of our splenic CD11b^+^ cells were phenotypically similar to Mo-MDSC and PMN-MDSC for the Ly6G^hi^Ly6C^+^ neutrophils. Mo-MDSC-like markers were expressed by most of the Ly6C^hi^ and Ly6C^lo/int^ monocytes, suggesting that these two splenic populations represent a mixture of various intermediate differentiation stages. MDSC have been best studied in the context of cancer, where they are known to inhibit T cell proliferation and/or function [24, 42, 43]. Recent literature, however, highlights their role in parasitic diseases as well [44]. *Heligmosomoides polygyrus*, for instance, induces MDSC capable of suppressing Th2 cell proliferation and IL-4 secretion, promoting therefore chronic infection [45]. In contrast, during experimental *L*. *major* infection, MDSC appear to be required for protective immunity even if they inhibit Th1 cell proliferation [46]; surprisingly, in this model, MDSC effector functions seem to be mouse-strain specific [47]. Our results indicate that myeloid cells purified from *L*. *donovani* infected mice at day 28 post infection are rather inhibitory. Indeed, they are able to significantly reduce IFNγ production of anti CD3/CD28-stimulated CD4 T cells. Interestingly, cells purified at day 14 post infection, before they acquire a MDSC-like phenotype, didn’t display any suppressive effect. At this time point of infection, mice have not yet developed severe splenomegaly, the splenic architecture is still intact [48], and HIF-1α expression in myeloid cell is very low (S2A and S2B Fig). This suggests that the splenic environment further shapes myeloid cell’s function to acquire inhibitory competence during the chronic phase of infection.

Suppression of Th1 cells was dependent upon HIF-1α expression. In fact, HIF-1α-deficient MDSC only displayed minor inhibitory functions even when purified from chronically infected mice. HIF-1α is known to enhance MDSC differentiation and effector functions in tumor immunology [4, 49]. MDSC mediate suppression through various mechanisms, such as upregulation of PD-L1, induction of IL-10, secretion of NO or ROS, or increased arginase activity [24]. In our model, we do not know what is responsible for suppressing Th1 responses. NO has been reported to inhibit T cell proliferation during *L*. *major* and *H*. *polygyrus* infections. In our case, HIF-1α-deficient MDSC-like cells expressed higher *Inos* mRNA levels than HIF-1α-sufficient, yet their inhibitory functions are significantly attenuated. Moreover, we didn’t see a substantial difference in IL-10 expression between *Cre*^*+*^ and *Cre*^*-*^ cells, suggesting that IL-10 may not play a major role. However, arginase expression was downregulated in HIF-1α-deficient myeloid cells compared to their HIF-1α-sufficient counterpart. In human cutaneous leishmaniasis, increased arginase activity has been associated to chronic infections [50, 51]; in these studies, neutrophils were the enzyme’s main source. Moreover, parasite-derived arginase was reported to contribute to the regulation of CD4 T cell exhaustion during experimental *L*. *major* infection [52]. It is thus possible that arginase may play a role in our model as well. Further investigations are needed to characterize the mechanism of suppression of MDSC-like myeloid cells in experimental VL.

Myeloid cells during *L*. *donovani* infection not only possess inhibitory capacities, but have also a propensity to be more permissive to *L*. *donovani* infection. Indeed, we found elevated mRNA levels for markers typically associated with the M2 macrophage phenotype, which is unable to kill the parasite [33]. Interestingly, HIF-1α conditional knockouts expressed significantly lower mRNA for M2-like markers during chronic experimental VL, suggesting that HIF-1α plays a major role in the induction of a M2-like phenotype. In a Lewis lung carcinoma model, polarization of tumor associated macrophages towards an M2-like phenotype was dependent on HIF-1α induced by tumor-derived lactic acid, a by-product of glycolysis [3]. In our model, splenocytes from infected *Hif* ^*flox/flox*^-*Cd11c-Cre*^-^ mice showed significantly higher intracellular lactate concentrations compared to *Cre*^*+*^ mice. It is thus possible that lactate contributes to HIF-1α stabilization in our model as well. We also noticed a lower glucose concentration in HIF-1α-deficient splenocytes. This may reflect a higher metabolic activity or a decreased capacity to import glucose into the cell, since HIF-1α induces the glucose transporter Glut-1expression [53].

Although our results are in agreement with Colegio et al [3], other groups have shown that HIF-1α promotes NOS2 in myeloid cells [28–30, 36] and is associated with M1-like macrophages. This discrepancy may be a reflection of the environment and the stimuli responsible for the stabilization of HIF-1α within the cells. In some infection models or in vitro experiments, HIF-1α appears to be mainly expressed by M1-like macrophages and to promote pathogen clearance [28, 30, 36, 54]; while in models of chronic inflammation, HIF-1α has a more immunosuppressive role [3, 49]. Under normoxic conditions, this transcription factor can be induced by inflammatory cytokines, TLR agonists, or directly by pathogens [55, 56] [8, 29, 57, 58]. In contrast, chronically inflamed tissues are generally hypoxic, acidic, hypoglycemic, and full of free oxygen radicals [59, 60]. Thus, depending on the model, HIF-1α stabilization occurs through very different pathways and this could lead to different outcomes. In our model, ex-vivo purified myeloid cells expressed higher iNOS mRNA levels in the absence of HIF-1α. This was not due to a compensatory upregulation of HIF-2α, which was only transiently higher at d14 p.i. in HIF-1α-deficient myeloid cells (S8 Fig). These results were confirmed using in vitro infection of BMM. Indeed, *L*. *donovani* strongly induced iNOS in HIF-1α deficient BMM, suggesting that this enzyme’s regulation in BMM may be manipulated by the parasite and that HIF-1α is somehow involved. However, the interpretation of these results could be tainted by the fact that some cells may escape recombination and that the interaction of HIF-1α ^+^ and HIF-1α^-^ cells may play a role in the total iNOS induction. Further investigations are definitely warranted to better understand iNOS regulation in *L*. *donovani* infected monocytes/macrophages and the role HIF-1α may have in this process.

Despite the fact that HIF-1α is involved in promoting endothelial cell proliferation [61] and Ly6C^hi^ monocytes contribute to red pulp vasculature remodelling [62], this transcription factor doesn’t seem to be the major player in tissue neovascularization and splenomegaly in experimental VL. Indeed, CD11c-HIF-1α conditional knockout and HIF-sufficient animals developed similar levels of splenomegaly and myeloid cells from both group of mice expressed similar levels of vascular endothelial growth factor (VGEF) mRNA.

To conclude, our results demonstrate that emergency myelopoiesis following *L*. *donovani* infection results in the output of monocytes primed in the bone marrow to acquire regulatory functions in a HIF-1α-independent manner. Once monocytes reach the spleen and start differentiating into macrophages or dendritic cells, the chronically inflamed splenic environment induces the stabilization of HIF-1α, which is then taking control over their functions. HIF-1α is responsible for the acquisition of MDSC-like functions by myeloid cells, and for lowering their leishmanicidal capacity. Because myeloid cell can also be produced locally in the spleen, it would be interesting to compare the function of bone marrow and splenic-derived monocytes. Finally, our study demonstrates how *L*. *donovani* exploits a physiological response to hypoxia to establish persistent infection.

## Material and methods

### Mice and parasites

C57BL/6-*Tg(OT-I)-RAG1*^*tm1Mom*^ mice were purchased from The Jackson Laboratory. Conditional *Hif-1α* knock-out in CD11c^+^ cells were generated as previously described [5]. All mice were housed at the INRS animal facility under specific pathogen-free conditions and used at 6–10 weeks of age.

*Leishmania donovani* (strain LV9) was maintained by serial passage in B6.129S7-*Rag1*^*tm1Mom*^ mice, and amastigotes were isolated from the spleens of infected animals. *Hif-1α Cd11c-Cre*^*+*^ mice and their littermates *Hif-1a*^flox/flox^-*Cd11c-Cre*^*-*^ were infected by injecting 2×10^7^ amastigotes intravenously via the lateral tail vein. Splenic parasite burdens were determined by examining methanol-fixed, Giemsa stained tissue impression smears [5]. Bone marrow parasite burden were calculated by limiting dilutions [5]. Data are presented as number of parasites present in the bone marrow of one femur and one tibia or as Leishman Donovan Units (LDU).

### Ethics statement

Experiments were carried out under protocols approved by the Comité Institutionel de Protection des Animaux of the INRS-Institut Armand-Frappier (1602–02, 1510–02). These protocols respect procedures on good animal practice provided by the Canadian Council on animal care.

### Flow cytometry

Myeloid cell responses in infected mice were analyzed by flow cytometry. Fc receptors were blocked by adding supernatant of 2.4G2–producing hybridomas for 5 min at 4°C to block to the homogenized splenocytes. Cells were then washed with FACS buffer and stained with the following antibodies: anti-MHCII FITC conjugated, anti-CD11c-APC (BD Biosciences), anti-CD11b Pacific Blue (PB), anti-Ly6C-Percp, anti-Ly6G-PE (Biolegend), anti-F4/80-PE-Cy7 (eBioscience), anti-IFNγR (eBioscience), and anti-CCR2-Alexa Fluor 700 (R&D Systems). The bone marrow (BM) was harvested by flushing tibias and femurs from the hind limbs in phosphate-buffered saline (PBS). Cells were passed through 25-gauge needles to obtain single cell suspensions. Single cell suspensions were prepared in PBS containing 0.1% bovine serum albumin (BSA) and 0.5mM ethylene-diamine-tetra-acetic acid (EDTA). To analyze adult BM progenitor cell populations, biotin-conjugated anti-lineage mAbs anti-CD3e (145-2C11), anti-CD11b (M1/70), anti-CD45/B220 (RA3-6B2), anti-Gr1 (RB6-8C5), and anti-Ter119 were used as the lineage mix. For secondary detection streptavidin conjugated to Brilliant Violet-500 was used. The hematopoietic stem progenitor cell (HSPC) population was analyzed by staining with PE anti-CD117 (c-Kit, 2B8; BD-Biosciences) and PE-Cy7 anti-Sca-1 (Ly6A/E, D7; BD-Biosciences) in addition to the lineage mix. Granulocyte-monocyte progenitor GMPs, were determined by staining with PE anti-CD41 (eBioscience), PerCP-Cy5.5 anti-CD16-32 (Biolegend) and Alexa-Fluor 647 anti-CD150 (TC15, BD Biosciences. 300,000 events were acquired on a BD LSRFortessa^TM^ cell analyzer (Becton Dickinson); analysis was performed using the FlowJo and/or FACSDiva software.

The expression of Total Reactive Oxygen Species (ROS) from infected mice was assessed using the (ROS) Assay Kit 520 nm kit (eBiosciences) following manufacturer’s instructions (catalogue number, 88-5930-74). In order to characterize ROS production in myeloid cell subpopulations, the antibodies previously described were added after fixation with 2% paraformaldehyde (PFA). Cells were acquired with a BD LSRFortessa^TM^ cell analyzer (Becton Dickinson).

Detection of hypoxia in the spleen of infected and naïve mice was performed using the Hypoxyprobe-RedAPC kit (Hypoxyprobe Inc., Burlington, MA) following manufacturer’s instructions. Cells were acquired with a BD LSRFortessa^TM^ cell analyzer (Becton Dickinson).

### Real-time PCR analysis

Real-time PCR (Stratagene mx3005p Real time PCR System) was used to analyze transcripts levels of HIF-1α, HPRT [5], TNF [63], Arg1, Fizz1, Mgl1, Mgl2 [3], and iNOS [64]. Total RNA was isolated using RNeasy (Qiagen) to perform real-time RT-PCR. cDNA was prepared using 500 ng of total RNA using High capacity cDNA Reverse Transcription kit (Bio Rad). Real time PCR was performed using standard cycle of amplification. Primers used to determine the relative gene fold expression by quantitative PCR (qPCR) are shown in Table 1.

**Table 1:** Primer sequences used in qPCR.

**Primers**	**Sequence**
***Hprt***	FP, 5′-GTTGGATACAGGCCAGACTTTGTTG-3′ RP, 5′-GATTCAACCTTGCGCTCATCTTAGGC-3′
***Hif-1α***	FP, 5′-TCACCTGCTGCTATACATTCAC-3′ RP, 5′-TCACCTGCTGCTATACATTCAC-3′
***Tnf***	FP, 5′-AGGGATGAGAAGTTCCCAAATG-3′ RP, 5′-GGCTTGTCACTCGAATTTTGAGA-3′
***Inos***	FP, 5′-CGAAACGCTTCACTTCCAA-3′ RP, 5′-TGAGCCTATATTGCTGTGGCT-3′
***Arg-1***	FP, 5′-CCACAGTCTGGCAGTTGGAAG-3′ RP, 5′-GGTTGTCAGGGGAGTGTTGATG-3′
***Fizz-1***	FP, 5′-CCTGCTGGGATGACTGCTA-3′ RP, 5′-TGGGTTCTCCACCTCTTCAT-3′
***Mgl-1***	FP, 5′-CAGAATCGCTT AGCCAATGTGG-3′ RP, 5′-TCCCAGTCCGTGTCCGAAC-3′
***Hif-2α***	FP, 5′-GGGAACACTACACCCAGTGC-3′ RP, 5′-TCTTCAAGGGATTCTCCAAGG-3′
***Glut-1***	FP, 5′-CGTGCTTATGGGTTTCTCCAAA-3′ RP, 5′-GACACCTCCCCCACATACATG-3′
***IL-10***	FP, 5′-AGGGTTACTTGGGTTGCCAA-3′ RP, 5′-CACAGGGGAGAAATCGATGA-3′

### In vitro bone marrow derived macrophage polarization and *L*. *donovani* infection

Macrophages were derived from the bone marrow of naïve mice in IMEM medium (Life Technologies) supplemented with 10% FBS, pen/strept, L-glutamine, and 15% L929 cell-conditioned medium as a source of colony-stimulating factor-1 (CSF-1). Cells were then left for 6 days at 37°C in a 5% CO_2_ incubator. For one set of experiments, BMM were washed and resuspended in supplemented DMEM. Cells were classically activated (M1) with 20 ng/ml of murine IFNγ (Peprotec), alternatively activated (M2) with 20 ng/ml of murine IL-4 (Peprotec), or infected with *L*. *donovani* amastigotes at a MOI of 1:10. BMM were then incubated for 24h and stained with anti-CD11b-BV421, iNOS-APC (Biolegend), and CD38-BV711 (eBioscience) or processed for qPCR analysis. Samples were acquired on a BD LSRFortessa cell analyzer (Becton Dickinson).

### Determination of lactate and glucose concentration

A million splenocytes from infected mice were washed with PBS and lysed with RIPA buffer (Sigma, catalogue number R0278-50). Intracellular lactate and glucose concentrations were measured using respectively a Lactate Assay Kit and Glucose Assay Kit (BioVision) as per manufacturer’s instructions. Samples were prepared as triplicates for the colorimetric lactate assay. The absorbance was measured at 570 nm using an xMark^TM^ microplate absorbance spectrophotometer (BioRad) immediately after preparation.

### CD4^+^ T cells inhibition test

Splenic CD4^+^ T cells were enriched from naïve mice using magnetic cell sorting (MACS) following manufacturer’s instructions (Miltenyi Biotec). The purity comprised between 90–95%. CD11b^+^ cells were purified using MACS from spleens of infected and naïve mice previously digested with collagenase D; the purity of the samples was 80–90%. Microtest 96 well plates (Sarstedt) were coated with/without 200μl of PBS containing 1 μg/ml anti-mouse CD3 (eBiosiences) and incubated for 90 min at 37°C. Plates were then washed twice with PBS and equilibrated for 15 min at 37°C with 100μl per well of RPMI-1640 medium (Life technologies), supplemented with 10% fetal bovine serum (FBS), pen/strep and L-glutamine. Th1 polarization was induced as follows: CD4^+^ T cells were seeded at 2 × 10^5^/well in anti-CD3-coated 96-well plates with anti-CD28 (2 μg/ml), rIL-12 (30 ng/ml), and rhIL2 (0.5 μg/ml) (eBiosciences). CD11b^+^ cells enriched as described above were added or not to the culture at a 1:1 ratio. Cells were then incubated at 37°C in a 5% CO_2_ incubator and 5 days later stimulated with phorbol 12-myristate 13-acetate (PMA)/ionomycin in the presence of Brefeldin A (BD Biosciences). Production of IFNγ was analyzed by FACS using anti-CD4-FITC, anti-CD3-PB, and anti-IFN_ϒ_-APC (BD-Bioscience). 50,000 events were acquired on a BD LSRFortessa cell analyzer and analyzed using the FlowJo software.

### Monocyte differentiation and in vitro *L*. *donovani* infection

Monocytes were derived from the bone marrow of naïve mice under hypoxia (2%) in IMEM medium (Life Technologies) supplemented with 10% FBS, pen/strept, L-glutamine, and 15% L929 cell-conditioned medium as a source of colony-stimulating factor-1 (CSF-1). Cells were then left for 3 days at 37°C in a hypoxia chamber. For one set of experiments, differentiated monocytes were washed and resuspended in supplemented DMEM without CSF-1 prior to a 2h-activation with 100 U/ml murine IFNγ (Peprotec); for another set of experiments, CSF-1 was left in the culture for the entire duration of the test. *L*. *donovani* amastigotes were stained with PKH67 (Sigma) following manufacturer’s instructions and added at a MOI of 1:10 for 1-24h under hypoxic condition. Cells were then stained with anti-CD11b-PB, Ly6C-PerCp, and CD11c-APC acquired on a BD LSRFortessa cell analyzer (Becton Dickinson) and Image stream (Amnis).

### Western blot analysis

Total cell protein extracts of CD11b^+^ cells purified by MACS from infected and naive mice were pooled and lysed in RIPA buffer (sigma Aldrich, Germany). *Cre*^*+*^ and *Cre*^*-*^ bone marrow-derived monocytes infected with *L*. *donovani* amastigotes were lysed as described above. Equal amounts of protein (15 μg) were fractionated by 10% SDS-PAGE. Monoclonal anti-HIF-1α antibody Hif-1α67 (Novus Biologicals, Littleton, CO, USA) was used for immunoblot assays. Blots were stripped and reprobed with a polyclonal antibody against β-actin to confirm equal protein loading [65]. Densitometric analysis was performed by spot densitometry using AlphaImager 3400 imaging software (Alpha Innotech Corporation) and normalized to ß-actin control. Values are presented as fold induction compared to the level in naive mice.

### Image stream flow cytometry

Monocytes were differentiated, treated, and stained as described above. After fixation with 2% PFA nucleus were stained with 4',6-diamidino-2-phénylindole (DAPI) and washed with PBS. Samples were then acquired on the ImageStreamX MarkII imaging cytometer (Amnis). The analysis was performed using the IDEAS software (Amnis).

### Immunohistochemistry

Freshly harvested spleens were snap frozen in OCT (Electron Microsopy Sciences, Hartfield, PA, USA) and stored at −80°C. Immunohistochemistry was performed on 8-μm frozen sections.

Tissue sections were fixed in 75% acetone and 25% ethanol (v/v) for 10 min at -20°C, rehydrated in PBS for 10 min at room temperature, and incubated for 1 hour with 5%-BSA in PBS supplemented with 2.4G2 supernatant (1:100). Slides were then incubated over night at 4°C with anti-CD11b-BV421 (BD Bioscience, 1:300), anti-B220-FITC (BioLegend, 1:500), and anti-CD169-A594 (BioLegend, 1:500). Tissue sections were then washed in PBS, mounted with Fluoromount-G (Electron Microscopy Sciences, Hatfield, PA, USA), and analyzed using a LSM780 confocal micoscope (Carl Zeiss, Oberkochen, Germany).

### Statistical analysis

Statistical analysis was performed using a multi-way ANOVA or Student’s t-test (only Figs 5 and 6), with p<0.05 considered significant. All experiments were conducted independently at least three times.

## Supporting information

S1 Fig *Hif*^*flox/flox*^-*Cd11c-Cre*^*+*^ and *Cre*^*-*^ mice were infected with *L*. *donovani* amastigotes and sacrificed at various time points of infection. (**A**) Graph represents the splenic parasite burden expressed as Leishman Donovan Units (LDU). (**B**) DNGR1 expression by conventional CD11c^hi^ splenic CD4^+^ (upper panel) and CD8^+^ (lower panel) DCs at d14 p.i. All data represent mean ± SEM of one of 4 independent experiments, n = 4.
(TIF)

S2 Fig Mice were infected with 2x10^7^ LV9 amastigotes intravenously. (**A**) Graph represents real-time PCR analysis of HIF-1α mRNA and (**B**) HIF-1α protein expression in splenic CD11c^**+**^ cells purified from *Hif*^*flox/flox*^-*Cd11c-Cre*^*+*^ and *Cre*^*-*^ mice at various time points after infection.
(TIF)

S3 Fig Mice were infected with 2x10^7^ LV9 amastigotes intravenously. Representative FACS plot for Ly6G^hi^ neutrophils **(A)** and F4/80^+^ cells **(B)** in *Hif-1α*^*flox/flox*^*Cd11c-Cre*^*-*^ (left panels) and *Hif-1α*^*flox/flox*^*Cd11c-Cre*^*+*^ mice (right panels).
(TIF)

S4 Fig Mice were infected with 2x10^7^ LV9 amastigotes intravenously. Representative FACS plot for CCR2^+^ F4/80^+^ Ly6C^hi^ **(A)** and Ly6C^low/int^ **(B)** monocytes in *Hif-1α*^*flox/flox*^*Cd11c-Cre*^*-*^ (left panels) and *Hif-1α*^*flox/flox*^*Cd11c-Cre*^*+*^ mice (right panels). **(C)** Representative FACS plot for Ly6C^hi^ monocyte expressing MHCII^+^ in *Hif-1α*^*flox/flox*^*Cd11c-cre*^*-*^ (left panels) and *Hif-1α*^*flox/flox*^*Cd11c-Cre*^*+*^ mice (right panels). **(D)** Representative FACS plot for surface expression of Ly6C on CD11b^hi^ Ly6G^hi^ neutrophils in *Hif-1α*^*flox/flox*^*Cd11c-Cre*^*-*^ (left panels) and *Hif-1α*^*flox/flox*^*Cd11c-Cre*^*+*^ mice (right panels).
(TIF)

S5 Fig Mice were infected with 2x10^7^ LV9 amastigotes intravenously. (A) Representative histograms for total ROS production at various time points of infection in *Hif-1α*^*flox/flox*^*Cd11c-Cre*^*-*^ and C*re*^*+*^ mice. **(B-C)** Representative FACS plots for ROS expression in Ly6G^hi^ neutrophils **(B)** and Ly6C^hi^ monocytes **(C)** from *Hif-1α*^*flox/flox*^*Cd11c-Cre*^*-*^ (left panels) and *Hif-1α*^*flox/flox*^*Cd11c-Cre*^*+*^ mice (right panels).
(TIF)

S6 Fig **(A)** Splenocytes from naïve and *L*. *donovani* infected mice (d28 p.i.) were stained with hypoxyprobe and analyzed by FACS. (**B**) Western Blot analysis of HIF-1α expression in infected bone marrow-derived monocytes. Monocytes were derived under hypoxia for three days from the bone marrow of naïve *Hif*^*flox/flox*^-*Cd11c-Cre*^*+*^ and *Cre*^*-*^. M-CSF was then removed from the medium and cells were infected with fluorescently-labelled *L*. *donovani* amastigotes prior to activation or not with IFNγ. (The infection was monitored for 1h, 12h and 24h. (A) Representative FACS plots for LV9^+^Ly6C^hi^ **(C)** and LV9^+^ Ly6C^low/int^ moncoytes **(D)**.
(TIF)

S7 Fig Monocytes were derived under hypoxia for three days from the bone marrow of naïve *Hif*^*flox/flox*^-*Cd11c-Cre*^*+*^ and *Cre*^*-*^. Cells were then infected with fluorescently-labelled *L*. *donovani* amastigotes prior to activation or not with IFNγ; M-CSF was kept in the medium. The infection was monitored for 12 and 24h. Representative FACS plots for LV9^+^Ly6C^hi^ **(A)** and LV9^+^ Ly6C^low/int^ moncoytes **(B)**.
(TIF)

S8 Fig Real-time PCR analysis of mRNA expression levels *Hif2α* in splenic CD11c^+^ cells purified from infected *Hif*^*flox/flox*^-*Cd11c-Cre*^*+*^ and *Cre*^*-*^ mice at various time points after infection. (TIF)
