# Supplementary figures and images for: HIF-1α is a key regulator in potentiating suppressor activity and limiting the microbicidal capacity of MDSC-like cells during visceral leishmaniasis
Source: PLoS Pathog. 2017 Sep 11;13(9):e1006616. doi: 10.1371/journal.ppat.1006616 (PMC5608422; doi:10.1371/journal.ppat.1006616)

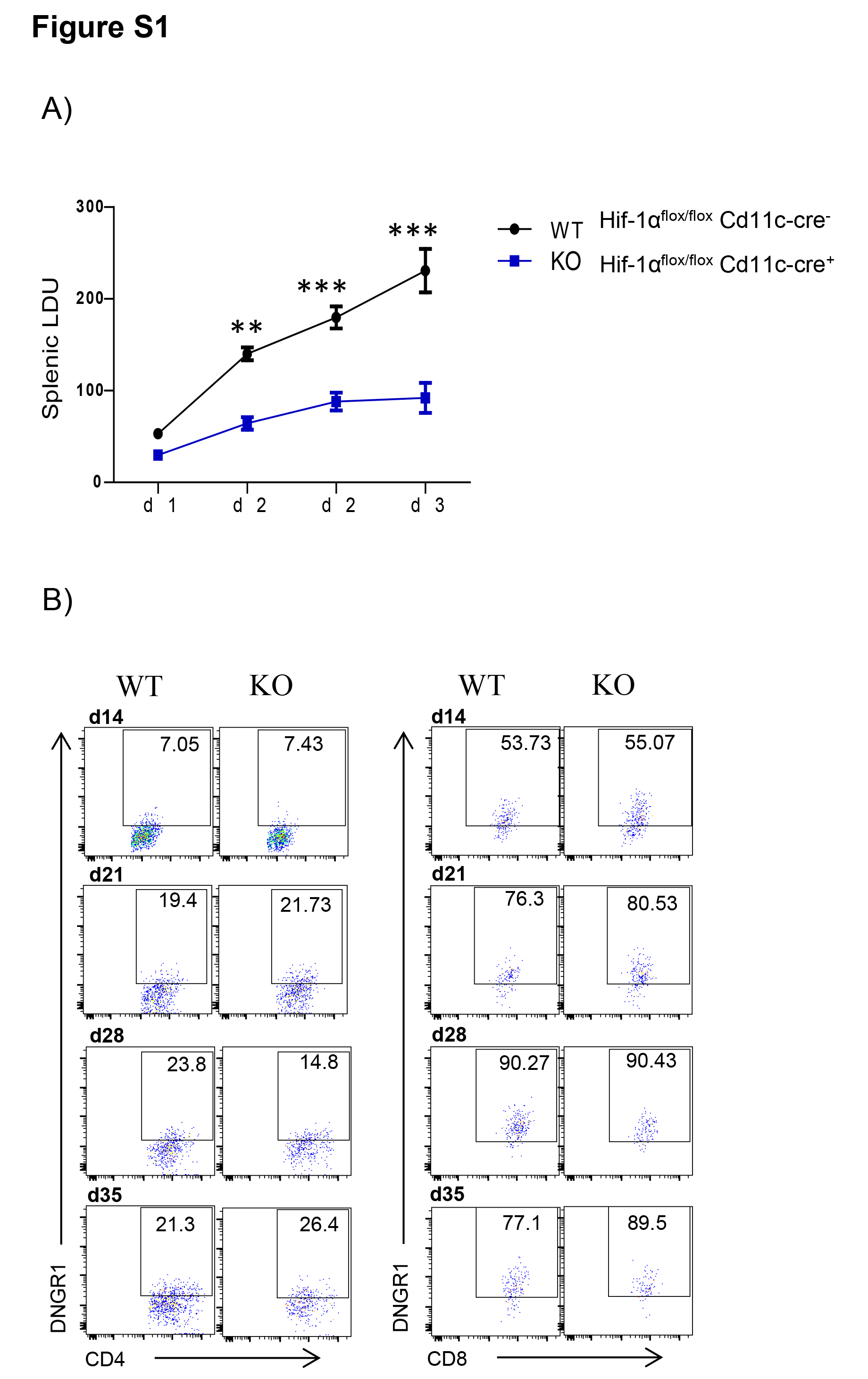

Supplement: S1 Fig — (A) Graph represents the splenic parasite burden expressed as Leishman Donovan Units (LDU). (B) DNGR1 expression by conventional CD11chi splenic CD4+ (upper panel) and CD8+ (lower panel) DCs at d14 p.i. All data represent mean ± SEM of one of 4 independent experiments, n = 4. (TIF) [file ppat.1006616.s001.tif]

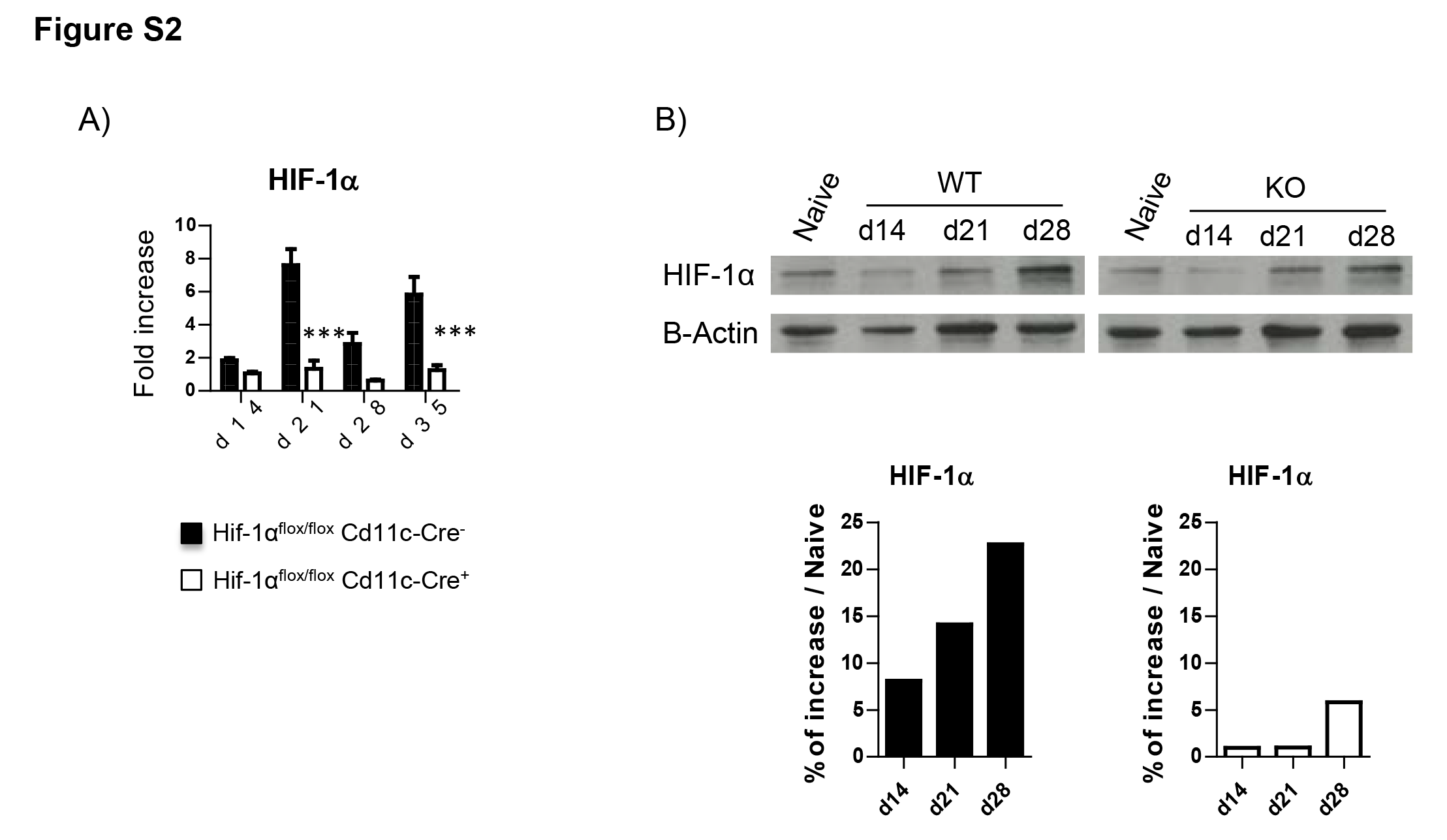

Supplement: S2 Fig — (A) Graph represents real-time PCR analysis of HIF-1α mRNA and (B) HIF-1α protein expression in splenic CD11c+ cells purified from Hifflox/flox-Cd11c-Cre+ and Cre- mice at various time points after infection. (TIF) [file ppat.1006616.s002.tif]

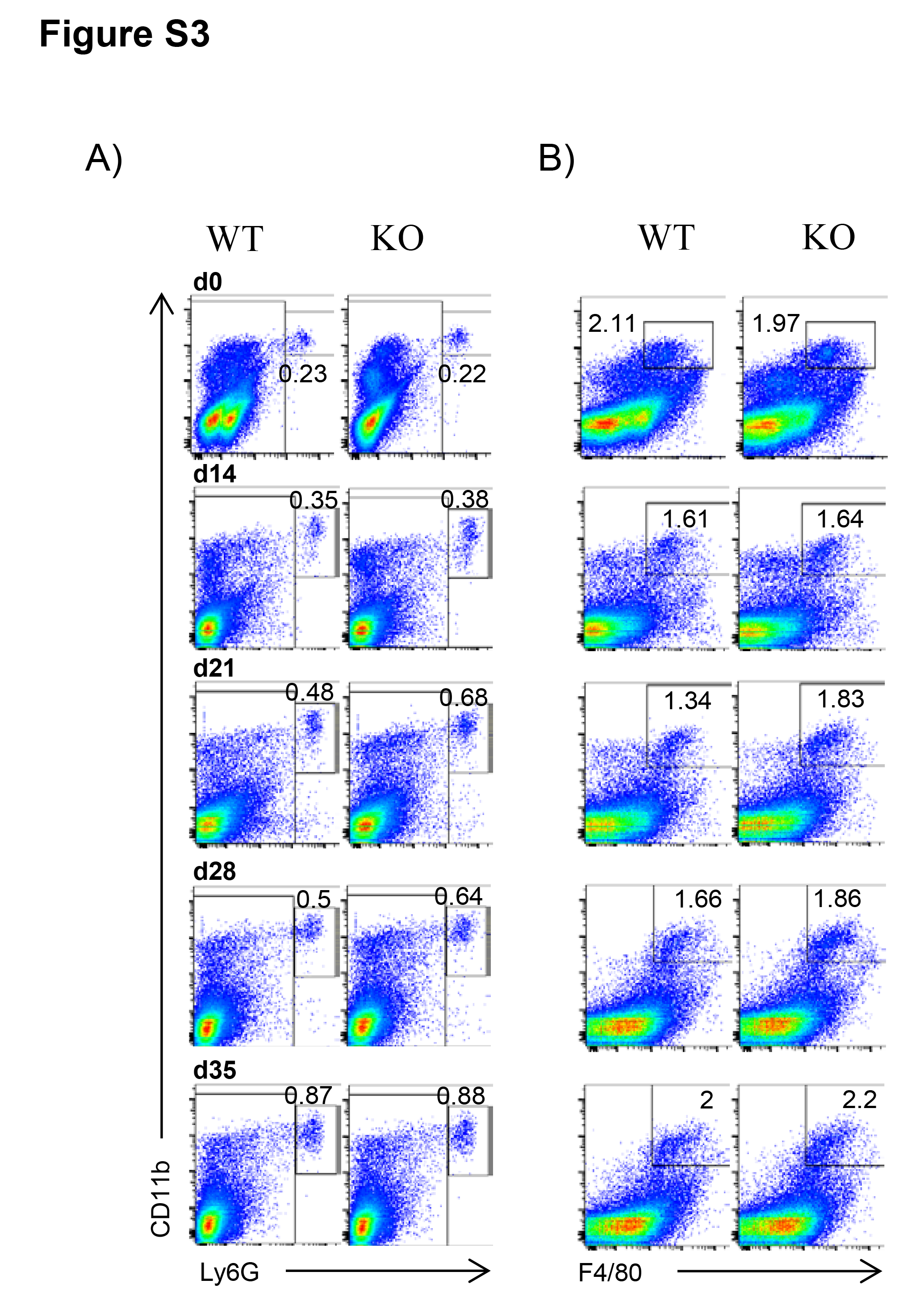

Supplement: S3 Fig — Representative FACS plot for Ly6Ghi neutrophils (A) and F4/80+ cells (B) in Hif-1αflox/floxCd11c-Cre- (left panels) and Hif-1αflox/floxCd11c-Cre+ mice (right panels). (TIF) [file ppat.1006616.s003.tif]

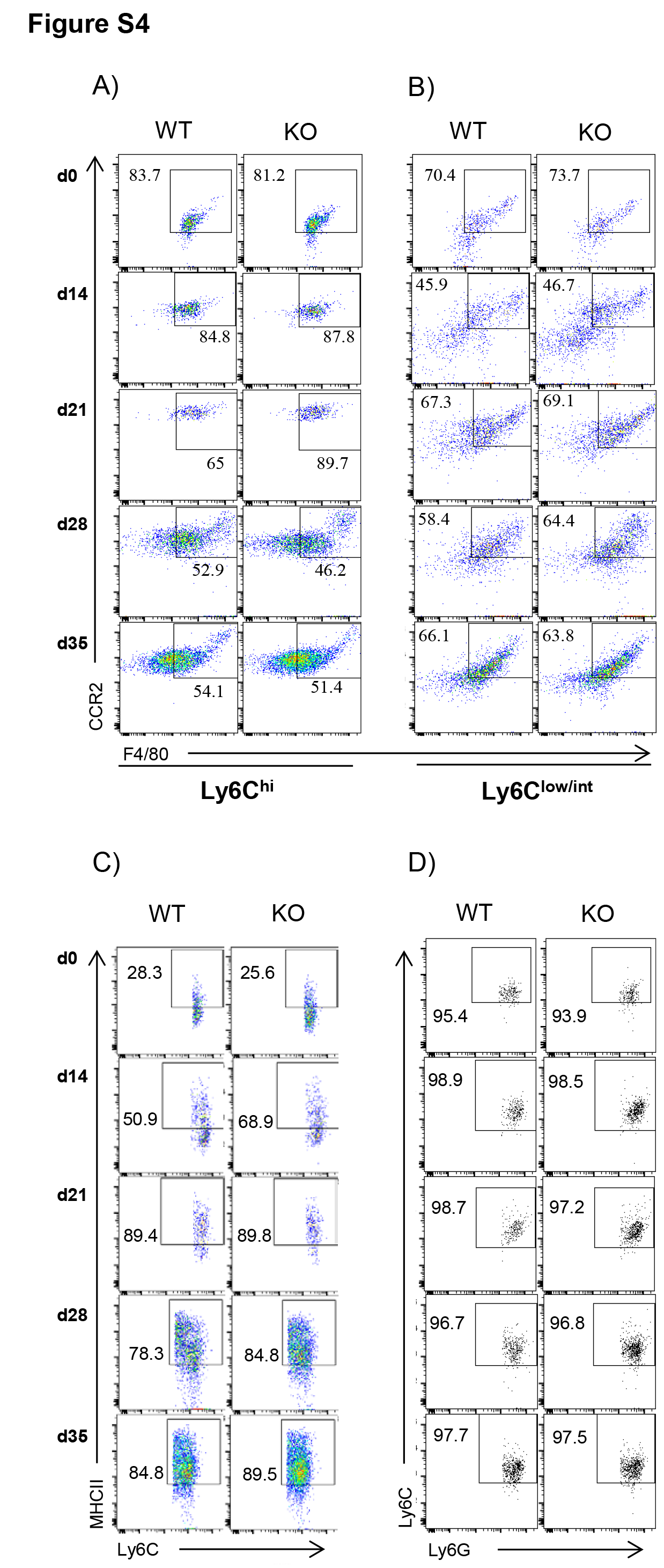

Supplement: S4 Fig — Representative FACS plot for CCR2+ F4/80+ Ly6Chi (A) and Ly6Clow/int (B) monocytes in Hif-1αflox/floxCd11c-Cre- (left panels) and Hif-1αflox/floxCd11c-Cre+ mice (right panels). (C) Representative FACS plot for Ly6Chi monocyte expressing MHCII+ in Hif-1αflox/floxCd11c-cre- (left panels) and Hif-1αflox/floxCd11c-Cre+ mice (right panels). (D) Representative FACS plot for surface expression of Ly6C on CD11bhi Ly6Ghi neutrophils in Hif-1αflox/floxCd11c-Cre- (left panels) and Hif-1αflox/floxCd11c-Cre+ mice (right panels). (TIF) [file ppat.1006616.s004.tif]

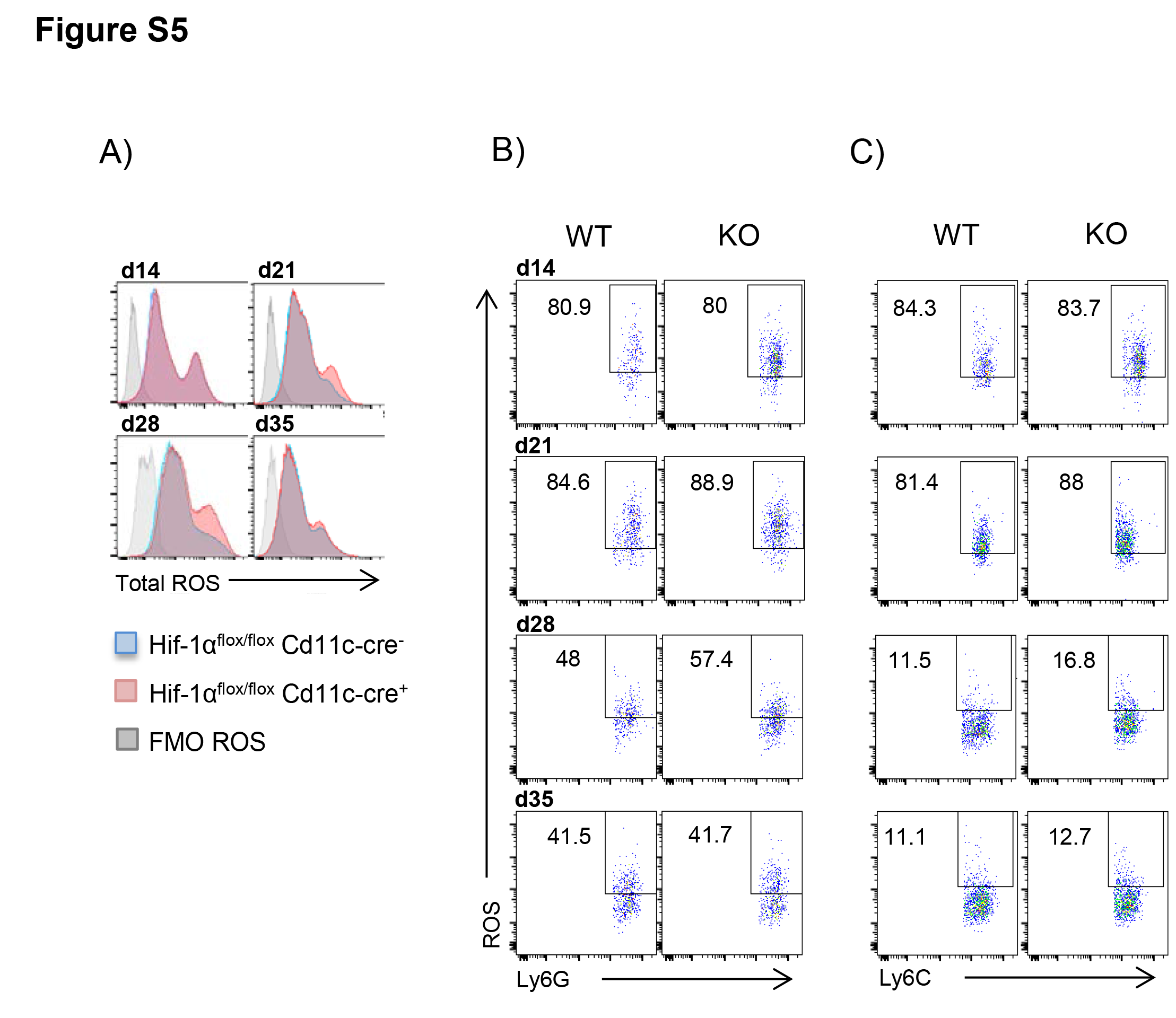

Supplement: S5 Fig — (A) Representative histograms for total ROS production at various time points of infection in Hif-1αflox/floxCd11c-Cre- and Cre+ mice. (B-C) Representative FACS plots for ROS expression in Ly6Ghi neutrophils (B) and Ly6Chi monocytes (C) from Hif-1αflox/floxCd11c-Cre- (left panels) and Hif-1αflox/floxCd11c-Cre+ mice (right panels). (TIF) [file ppat.1006616.s005.tif]

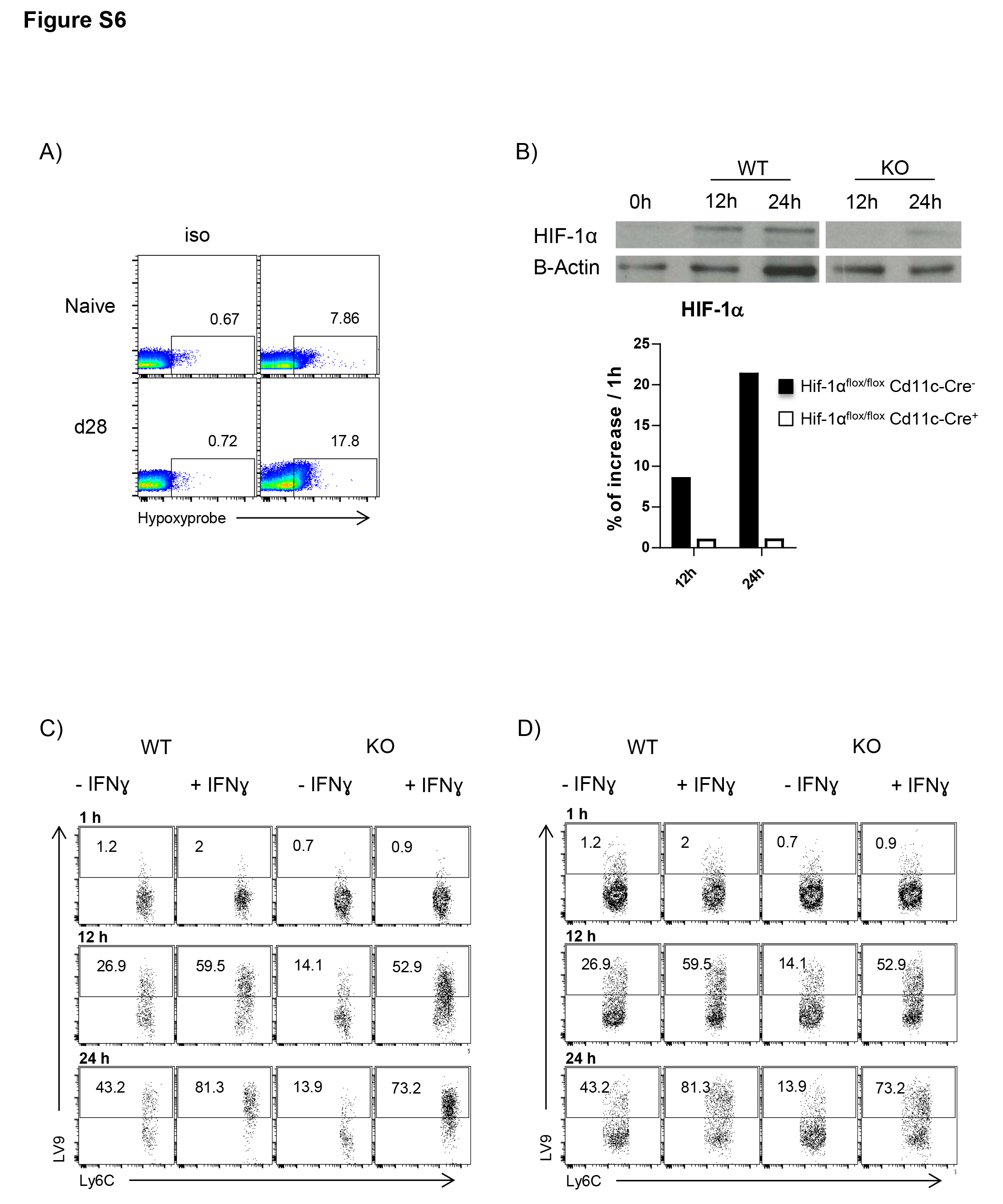

Supplement: S6 Fig — (A) Splenocytes from naïve and L. donovani infected mice (d28 p.i.) were stained with hypoxyprobe and analyzed by FACS. (B) Western Blot analysis of HIF-1α expression in infected bone marrow-derived monocytes. Monocytes were derived under hypoxia for three days from the bone marrow of naïve Hifflox/flox-Cd11c-Cre+ and Cre-. M-CSF was then removed from the medium and cells were infected with fluorescently-labelled L. donovani amastigotes prior to activation or not with IFNγ. (The infection was monitored for 1h, 12h and 24h. (A) Representative FACS plots for LV9+Ly6Chi (C) and LV9+ Ly6Clow/int moncoytes (D). (TIF) [file ppat.1006616.s006.tif]

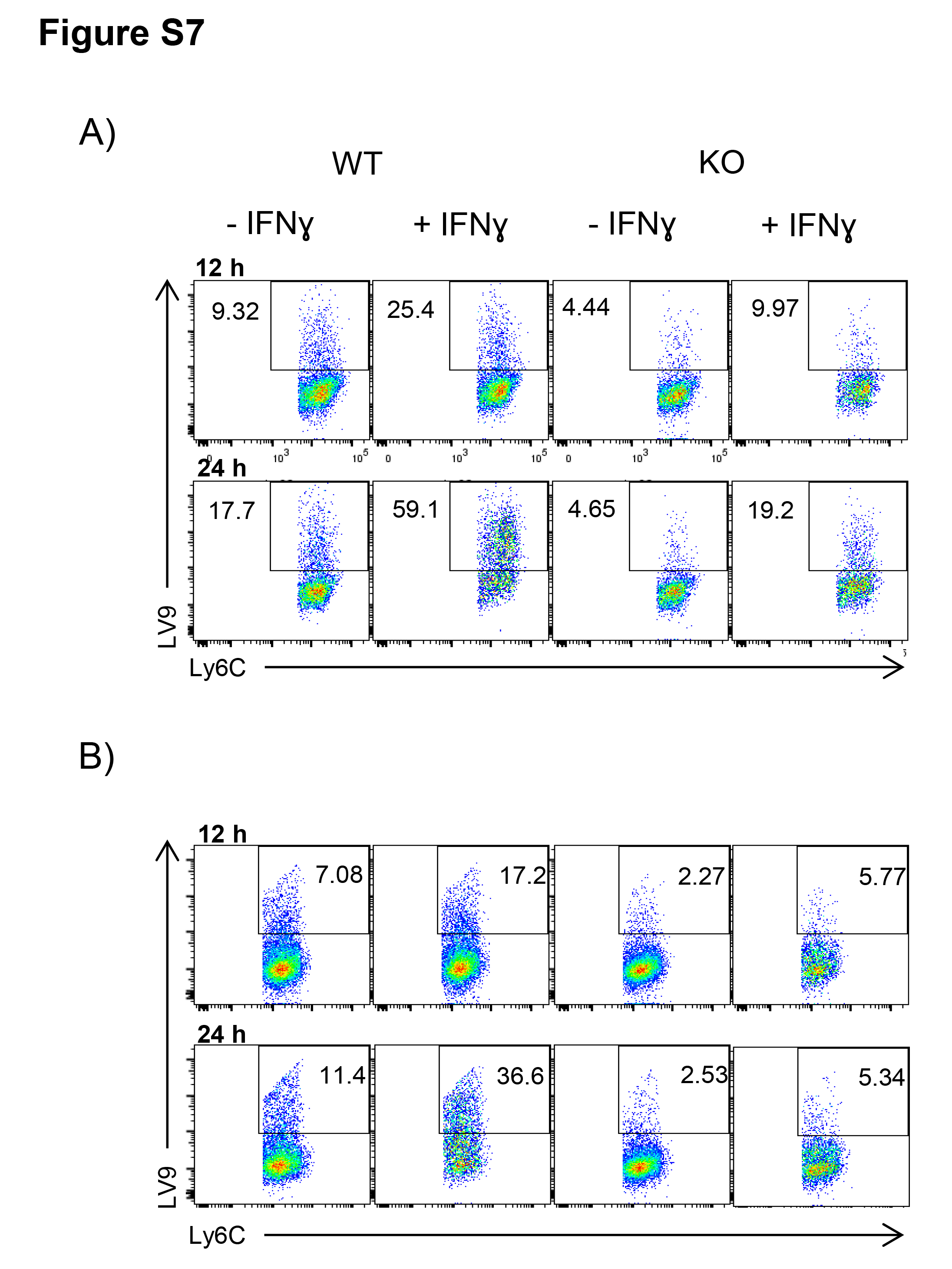

Supplement: S7 Fig — Cells were then infected with fluorescently-labelled L. donovani amastigotes prior to activation or not with IFNγ; M-CSF was kept in the medium. The infection was monitored for 12 and 24h. Representative FACS plots for LV9+Ly6Chi (A) and LV9+ Ly6Clow/int moncoytes (B). (TIF) [file ppat.1006616.s007.tif]

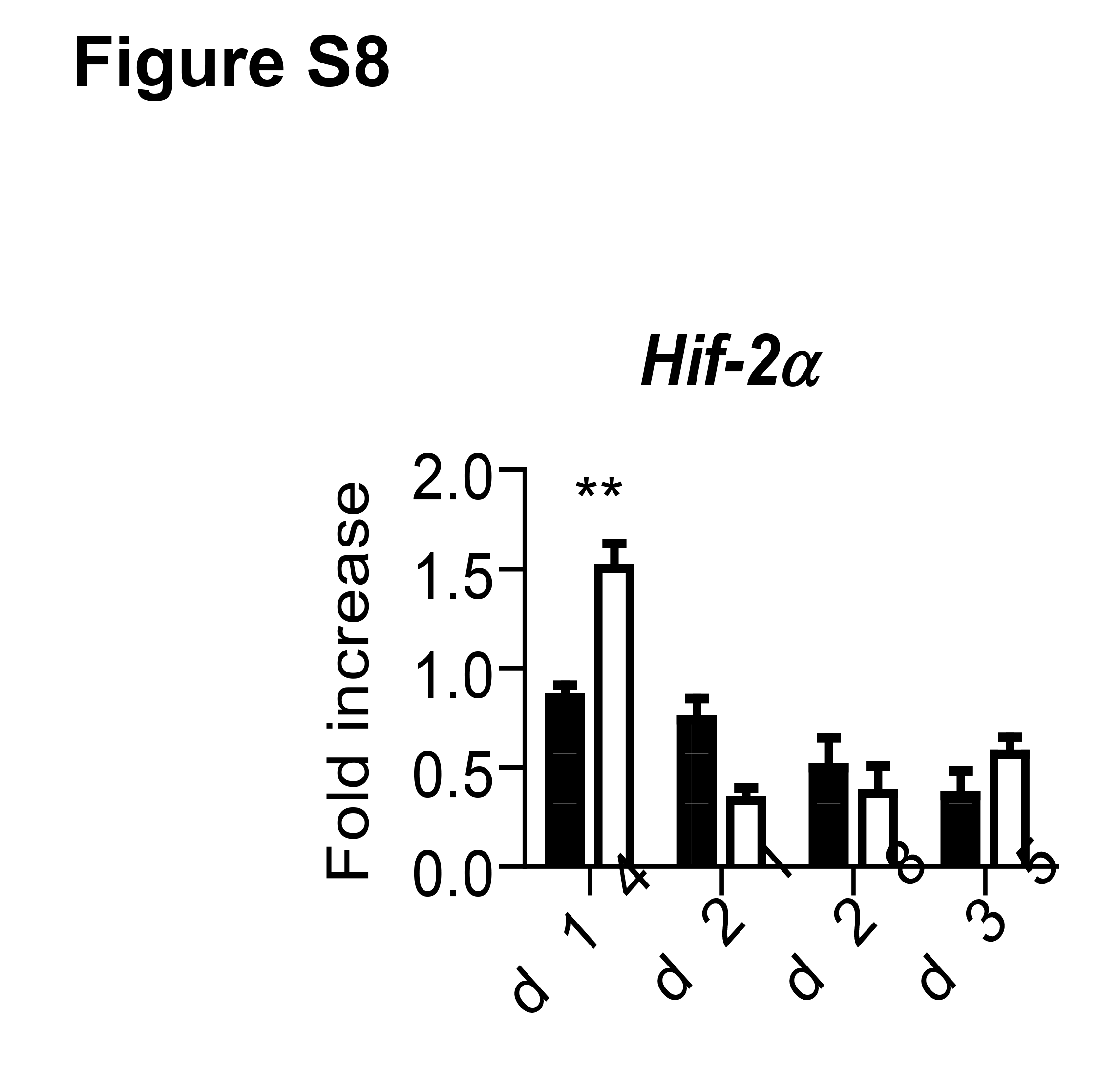

Supplement: S8 Fig — (TIF) [file ppat.1006616.s008.tif]
